# The role of intracellular signaling in the stripe formation in engineered *Escherichia coli* populations

**DOI:** 10.1371/journal.pcbi.1006178

**Published:** 2018-06-04

**Authors:** Xiaoru Xue, Chuan Xue, Min Tang

**Affiliations:** 1 School of Mathematics and Institute of Natural Sciences, Shanghai Jiao Tong University, Shanghai, China; 2 Department of Mathematics, Ohio State University, Columbus, Ohio, United States of America; UNITED KINGDOM

## Abstract

Recent experiments showed that engineered *Escherichia coli* colonies grow and self-organize into periodic stripes with high and low cell densities in semi-solid agar. The stripes develop sequentially behind a radially propagating colony front, similar to the formation of many other periodic patterns in nature. These bacteria were created by genetically coupling the intracellular chemotaxis pathway of wild-type cells with a quorum sensing module through the protein CheZ. In this paper, we develop multiscale models to investigate how this intracellular pathway affects stripe formation. We first develop a detailed hybrid model that treats each cell as an individual particle and incorporates intracellular signaling via an internal ODE system. To overcome the computational cost of the hybrid model caused by the large number of cells involved, we next derive a mean-field PDE model from the hybrid model using asymptotic analysis. We show that this analysis is justified by the tight agreement between the PDE model and the hybrid model in 1D simulations. Numerical simulations of the PDE model in 2D with radial symmetry agree with experimental data semi-quantitatively. Finally, we use the PDE model to make a number of testable predictions on how the stripe patterns depend on cell-level parameters, including cell speed, cell doubling time and the turnover rate of intracellular CheZ.

## Author summary

One of the central problems in biology is to understand the underlying mechanisms responsible for spatial pattern formation in complex systems. This is a difficult task because the essential mechanisms for pattern formation often involve multiple space and time scales and are often buried in overwhelmingly complex physiological details. Recently, synthetic biology has made it possible to investigate strategies of pattern formation in relatively simpler, but still complex, systems. Here we develop multiscale models to help explain the role of intracellular signaling in the formation of stripe patterns in engineered *E. coli* colonies.

## Introduction

Understanding the formation of regularly spaced structures, such as vertebrate segments, hair follicles, fish pigmentation and animal coats, is a fundamental problem in developmental biology [1–7]. These patterns involve the complex interaction of intracellular signaling, cell-cell communication, cell growth and cell migration. The overwhelmingly complex physiological context usually makes it difficult to uncover the interplay of these mechanisms. Synthetic biology has recently been used to extract essential components of complex biological systems and examine potential strategies for pattern formation [8–11].

**Fig 1:**
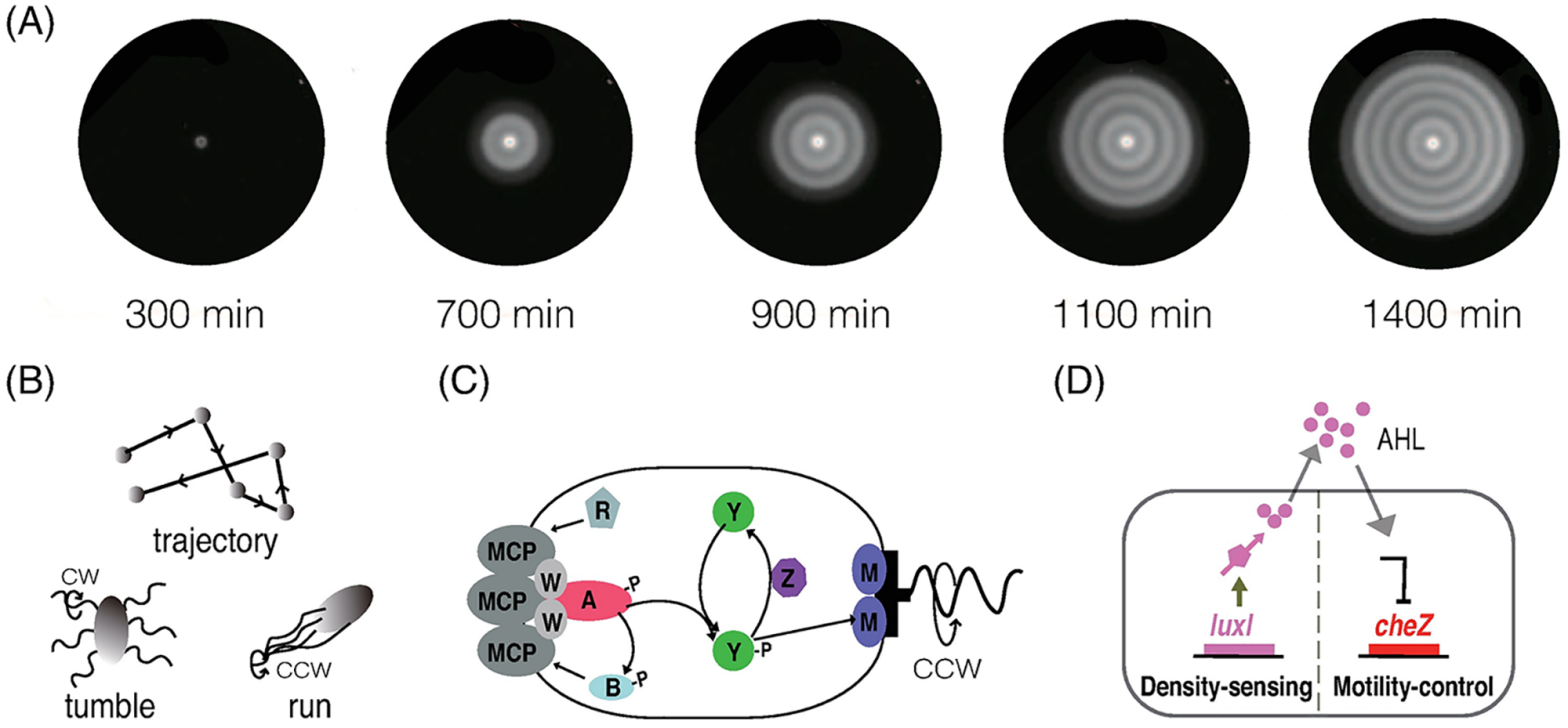
Sequential stripe formation in an engineered *E. coli* colony. (A) Concentric stripe patterns formed in experiments. Scale bar: 1 cm. (B) Run-and-tumble movement. (C)The intracellular chemotaxis pathway of *E. coli*. MCP is the transmembrane receptors. The letters in the figure represents the corresponding proteins involved in chemotaxis, e.g., A represents CheA. (D) The quorum-sensing module. (A), (D) Reproduced from Fig. 1 in Liu et al, Science, Vol 334, 238–241, 2011 [12].

One of these problems relate to the bacterium *Escherichia coli*. Recently in [12], the chemotaxis signaling pathway of *E. coli* has been engineered and coupled with a quorum sensing module, leading to cell-density suppressed cell motility. When a suspension of the engineered cells is inoculated at the center of a petri dish with semi-solid agar and rich nutrient, the colony grows, moves outward and sequentially establishes rings or “stripes” with a high density of cells behind the colony front (Fig 1A). These spatial patterns form in a strikingly similar way as many periodic patterns in other biological systems. When the maximum density of the motile front reaches a threshold, an immotile zone is nucleated. The immotile zone then absorbs bacteria from its neighborhood to expand, forming alternating high and low density zones. These patterns do not form when using wild-type *E. coli*; instead, the colony simply expands outward and forms a uniform lawn. The goal of this paper is to use mathematical models to elucidate the underlying mechanisms for this pattern formation, with a special focus on the roles of intracellular signaling.

*E. coli* is an enteric gram-negative bacterium that moves by alternating forward-moving “runs” and reorienting “tumbles”. It has 6-8 flagella on its surface that can rotate either clockwise (CW) or counterclockwise (CCW) (Fig 1B). If the majority of its flagella rotate CCW they form a bundle and push the cell to run forward with a speed ∼ 10 − 30*μ*m/s. If some flagella rotate CW they fly apart and the cell tumbles in place. *E. coli* can bias its movement in response to external chemical signals, e.g, towards locations with higher concentration of chemoattractant or lower concentration of repellent, which is called chemotaxis. The molecular mechanism of *E. coli* chemotaxis is summarized in Fig 1C. The transmembrane chemoreceptors (denoted as MCP) form stable ternary complexes with the intracellular signaling proteins CheA and CheW. CheA is an auto-kinase and also a kinase for the response regulators CheY and CheB. The activity of CheA depends on the ligand-binding state of the receptor complex as well as its methylation level: attractant-binding reduces CheA activity and methylation increases it. The phosphorylated form CheYp binds to the flagella motor and increases the probability of clockwise (CW) rotation. On the other hand, CheBp and CheR change the methylation state of the receptor at a slower rate: CheR methylates it and CheBp demethylates it. Upon attractant binding, CheA activity is reduced immediately, leading to lower CheYp and CheBp. Then a shift of the methylation-demethylation cycle gradually restores CheA activity on a slower time scale.

In [12], the quorum-sensing module of bacterium *Vibrio fischeri* was embedded into *E. coli* and used to control the transcription of *cheZ* (Fig 1D). The engineered cell synthesizes and secretes acyl-homoserine lactone (AHL), a small molecule that is freely diffusible across the cell membrane and degrades rapidly. At high concentrations, AHL suppresses the transcription of *cheZ* in an ultra-sensitive manner. If *cheZ* is suppressed, CheZ protein becomes diluted as the cell grows and divides. Because CheZ is a dephosphorylation kinase of CheYp, a reduction of CheZ protein can immediately lead to higher CheYp concentration and thus more persistent tumbles of the cell. This, in turn, causes changes to the chemoreceptors as well as to other proteins involved in chemotaxis, and triggers a non-classic chemotactic cellular response. To quantify the effect of AHL in single cell movement, one must take into account the whole chemotaxis pathway as well as CheZ turnover.

A phenomenological PDE model was used to explain the pattern formation process in [12] and a simplified version was analyzed in [13]. The model consists of a system of reaction-diffusion equations for the cell density, AHL and nutrient concentrations. The diffusion rate of the cell population is assumed to be a switch-like function of the local AHL concentration. Since the whole chemotaxis pathway is involved in the pattern formation process, it is unclear how cell movement can be reduced to an anisotropic (or cross) diffusion process. Moreover, the model does not address the role of intracellular signaling in stripe formation and cannot be used to understand how the spatial structure of the high-density and low-density regions depends on cell-level parameters.

To address these questions, we first developed a hybrid model for the stripe formation that accounts for the behavior of individual cells. The model starts with a detailed description of intracellular signaling, single cell movement and cell division. This individual-based component is then coupled with reaction-diffusion equations for AHL and nutrient concentrations. The multiscale nature of this model allows us to explore the relations between cellular processes on a time scale of seconds to minutes and population dynamics on a time scale of hours. Simulations of our hybrid model showed the same stripe patterns as observed in experiments, but they are very time-consuming due to the large number of cells involved in the pattern formation process. If cells double every 30 minutes, then during a typical time period for pattern formation, e.g. 10 hours, the population size can grow 2^20^ ≈ 10^6^ times.

To overcome this computational challenge, we then derived a macroscopic PDE for the cell density from the hybrid model, using asymptotic analysis and moment closure methods. Parameters of the PDE model are fully determined using parameters of the hybrid model. Numerical comparisons of the hybrid model and the PDE model showed quantitative agreement in 1D under biologically-relevant parameter regimes. This justifies using the PDE model as a quantitative and predictive tool to understand the relation between population patterning and cellular dynamics.

We then used our PDE model to investigate how concentric stripe patterns change when cells are subject to other chemicals or mutations as discussed in [12]. Numerical simulations of our PDE model in 2D with radial symmetry agree with experimental data semi-quantitatively. Finally, we used our PDE model to make a number of predictions on how stripe formation depends on cell-level parameters. Specifically, we investigated how the colony front speed, the wavelength of the spatial pattern and the structure within a single spatial element depend on the individual cell speed, cell doubling time as well as the rate of CheZ turnover. Our simulations suggested that the individual cell speed and the cell doubling time primarily affect the colony front speed and the pattern wavelength, while the the turnover rate of CheZ mainly affects the spatial structure of each stripe.

## Methods

**Fig 2:**
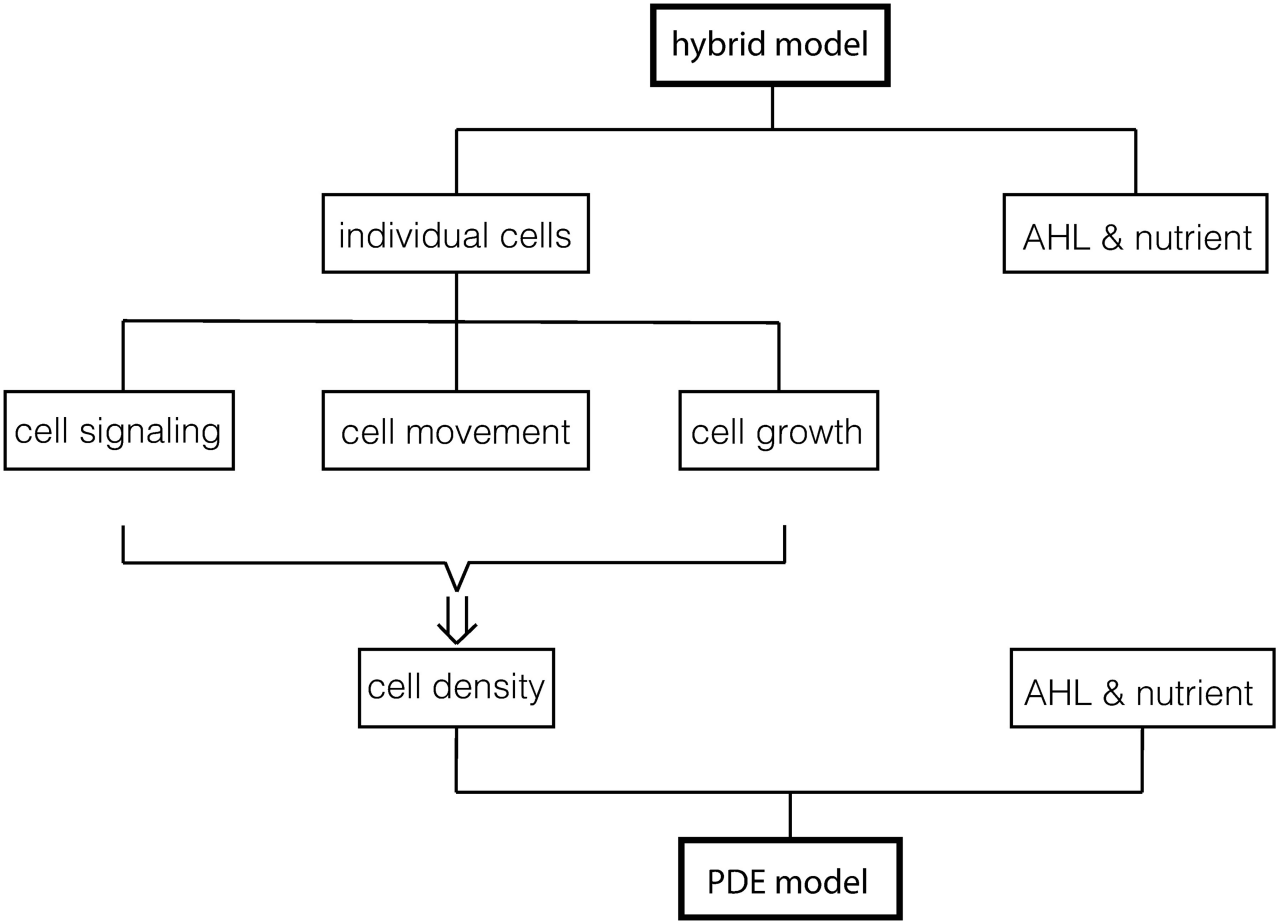
The structure of the hybrid model and the PDE model. The hybrid model includes an individual-based description for the cell dynamics, including intracellular signaling, cell movement and cell growth. The cell density equation (Eq 17) in the PDE model is derived from the probability density distribution for individual cells. The derivation is included in S2 Text. Both models use reaction-diffusion equations for the concentrations of the quorum-sensing signal AHL denoted by *h*(**x**, *t*) and nutrient denoted by *n*(**x**, *t*).

We describe two mathematical models: hybrid model and PDE model in this section. The structure of these models and their relation are shown in Fig 2. The initial and boundary conditions are described in Results.

### Hybrid model

Each cell is described as an individual particle with location **x**^*i*^, velocity **v**^*i*^, and internal state **y**^*i*^. The superscript *i* is the index for the cell. Cell signaling is modeled by an internal ODE system for **y**^*i*^. Cell movement is modeled by a velocity jump process in which transition rates are functions of **y**^*i*^. Cell growth is implemented by random creation of new daughter cells from mother cells. The cell dynamics is then coupled with reaction-diffusion equations for *h*(**x**, *t*) and *n*(**x**, *t*). A similar type of model was used to model pattern formation in the slime mold *Dictyostelium discoideum* in [14]. Details of each component are given below. For simplicity of notation, we omitted the superindex *i* below.

#### Intracellular signaling described by a system of ODEs

We adopt the mathematical model for *E. coli* chemotactic signaling described in [15], and extend it to include the temporal dynamics of total *CheZ* protein in response to extracellular *AHL*. The model in [15] is a simplified form of the model derived in [16]. These models are based on the detailed biochemistry of the signaling network and are derived rigorously from mass action kinetics and asymptotic analysis.

We denote the total concentration of CheZ protein, i.e., the sum of CheZ and CheZ_p_, by *z*(*t*). The dynamics of *z* is governed by protein production due to transcription and translation as well as dilution due to cell growth. Let *V*(*t*) be the volume of a cell and *k*_*V*_ be its growth rate. Then between cell divisions we have *V*′ = *k*_*V*_ *V*. If there is no production of CheZ protein, *Vz* remains constant and

$$z^{\prime }=-V^{\prime }z/V=-k_{V}z.$$

If we assume that the production rate of *CheZ* is constant in wild-type cells, then

$$z^{\prime }=k_{V}\left(Z_{w}-z\right),$$

where *Z*_*w*_ is the steady state of *z*. For the stripe-forming cells created in [12], high concentration of *AHL* can indirectly suppress the transcription of *cheZ*, resulting in a sharp decrease of *CheZ* (Fig 1C). To model this effect, we take

$$\begin{array}{c} z^{\prime }=g\left(z,h\right)=\{\begin{array}{cccc} & k_{V}\left(Z_{w}-z\right), & & \;\text{if}\;h<h_{0},\\ & -k_{V}z, & & \;\text{if}\;h\geq h_{0}, \end{array} \end{array}$$ (1)

where *h*_0_ is the threshold AHL level for the suppression of *cheZ*. Our simulations show similar results when a smooth interpolation of *g*(*z*, *h*) was used instead, but the transition of the two states has to be sharp.

We next couple *CheZ* dynamics with the rest of the chemotactic signaling pathway. Denote the mean methylation level of the chemoreceptors by *m*. Based on [15], the equation of *m* is governed by the methylation and demethylation reactions mediated by CheR (*R*) and CheB_*p*_ (*B*_*p*_) as

$$\begin{array}{c} \frac{\text{dm}}{dt}=f\left(m,z\right)=k_{R}R(1-A(m))-k_{Bp}B_{p}A\left(m\right). \end{array}$$ (2)

Here *A*(*m*) is the mean receptor activity:

$$\begin{array}{c} A\left(m\right)=\frac{1}{1+\exp\left[N_{r}\alpha_{0}\left(m_{0}-m\right)\right]}, \end{array}$$ (3)

where *m*_0_ = 1 is the reference methylation level, *α*_0_ = 1.7 measures how the free energy of the receptor complex depends on *m*, and *N*_*r*_ = 6 is the average number of nearest neighbors of the receptor functioning units. Note that *A*(*m*) increases with *m* and has a sharp transition near *m* = *m*_0_. In general, *A* is a function of both *m* and the ligand binding state of the receptors (see Eqn. 5.12 in [15]). The function *A* reduces to Eq (3) if there is no ligand-binding signal involved as in this context. *CheR* concentration *R* is given by

$$\begin{array}{c} R=\frac{R_{t}}{1+K_{R}T_{t}\left(1-A\left(m\right)\right)}. \end{array}$$ (4)

CheB_*p*_ concentration *B*_*p*_ is implicitly given by a system of algebraic equations of *B*_*p*_, *Y*_*p*_ (concentration of CheY_*p*_) and *T*_*p*_ (concentration of CheAp-associated receptors), as

$$\begin{array}{ccc} & k_{A}(T_{t}A\left(m\right)-T_{p})-k_{Y}YT_{p}-k_{B}BT_{p}=0, & \\ & k_{Y}YT_{p}-\mu_{Y}Y_{p}-k_{Z}ZY_{p}=0, & \\ & k_{B}BT_{p}-\mu_{B}B_{p}=0, & \end{array}$$ (5)

with

$$\begin{array}{c} \begin{array}{cc} & \;Y=\frac{Y_{t}-\left(1+K_{Z}Z\right)Y_{p}}{1+K_{Y}T_{p}},\;Z=\frac{z}{1+K_{Z}Y_{p}},\;B=\frac{B_{t}-\left(1+K_{Bp}T_{t}A\left(m\right)\right)B_{p}}{1+K_{B}T_{p}}. \end{array} \end{array}$$ (6)

Here the constants *T*_*t*_, *Y*_*t*_, *B*_*t*_ and *R*_*t*_ are the total concentrations of the corresponding proteins; *z* ≡ *Z*_*t*_ is total CheZ given by Eq (1); and the *k*’s, *K*’s, and *μ*’s are reaction rate constants. Eq (5) are derived from quasi-steady state approximations of *B*_*p*_, *Y*_*p*_ and *T*_*p*_ respectively, based on the fact that the chemical reactions involving *B*_*p*_, *Y*_*p*_ and *T*_*p*_ occur on a time scale much faster than the methylation and demethylation of the receptors. Eq (6) are derived from the conservation conditions [15, 16].

In summary, the ODE model for each cell is a pair of differential equations for the internal states *y* = (*z*, *m*) (CheZ concentration and receptor methylation level) coupled with several nonlinear equations for the intracellular chemotaxis protein concentrations. The parameter values for the model are summarized in Table 1. The parameters involved in chemotactic signaling are taken from [15, 16], please see references therein.

**Table 1:** Parameters for intracellular signaling. See [15, 16] for references.

Param.	Description	Values
*k*_*R*_	Methylation rate mediated by CheR	3.82 × 10^−2^ *s*^−1^
$k_{B_{p}}$	Demethylation rate mediated by CheB_*p*_	3.25*s*^−1^
*k*_*A*_	Phosphorylation rate of *T*_*p*_ mediated by CheA	100*s*^−1^
*k*_*Y*_	Dephosphorylation rate of *T*_*p*_ mediated by CheY	130*μM*^−1^ *s*^−1^
*k*_*B*_	Dephosphorylation rate of *T*_*p*_ mediated by CheB	7.5*μM*^−1^ *s*^−1^
*k*_*Z*_	Dephosphorylation rate of *Y*_*p*_ mediated by CheZ	8.45*μM*^−1^ *s*^−1^
*μ*_*Y*_	Degradation rate of *Y*_*p*_	0.1*s*^−1^
*μ*_*B*_	Degradation rate of *B*_*p*_	1*s*^−1^
*K*_*B*_	Association constant for CheB phosphorylation	0.25*μM*^−1^
$K_{B_{P}}$	Association constant for receptor demethylation	6.5*μM*^−1^
*K*_*R*_	Association constant for receptor methylation	0.15*μM*^−1^
*K*_*Y*_	Association constant for CheY phosphorylation	0.65*μM*^−1^
*K*_*Z*_	Association constant for CheYp dephosphorylation	1*μM*^−1^
*B*_*t*_	total concentration of CheB	2*μM*
*R*_*t*_	total concentration of CheR	0.3*μM*
*T*_*t*_	total concentration of CheT	5/3*μM*
*Y*_*t*_	total concentration of CheY	18*μM*
*Z*_*w*_	CheZ concentration of wild type *E. coli*	1.23*μM*
*h*_0_	AHL threshold (nondimensional)	0.25

#### Cell movement as a velocity-jump process with moving and resting states

*E. coli* cells move by alternating between two movement states, running and tumbling. The speed of running is about 10 − 30*μ*m/s, and during tumbling they stop immediately with almost no displacement. For wild-type cells, the average tumbling time (0.1*s*) is much shorter than the average running time (1*s*). For this reason, the tumbling time is frequently ignored in previous models [17, 18]. However, for the engineered cells we consider here, intracellular CheZ can be significantly lower than that of wild-type cells, leading to significantly longer tumbling. In this case cell tumbling cannot be naively ignored.

Based on the above considerations, we describe the movement of each cell as an independent velocity jump process with a moving state and a resting state. We assume cells can move in any directions with constant speed *s*_0_ = 20*μ*m/s. We denote the rate for a running bacterium to stop and tumble by λ and the rate for a tumbling cell to start running by *μ*, i.e.,

$$\text{Run}\overset{\lambda }{\underset{\mu }{⇌}}\text{Tumble}.$$

We further assume that a cell chooses a new direction randomly with equal probability after tumbling. Adding a slight directional persistence as observed in [19, 20] does not alter the main results of the paper.

The turning rates λ and *μ* depends on the intracellular CheYp level (*Y*_*p*_). We determine these rates using the following method that involves a voting process [21].

An *E. coli* cell has several flagella and each flagellar motor can rotate either clockwise (CW) or counter-clockwise (CCW). We denote the switching rates from CCW to CW by λ_*f*_ and from CW to CCW by *μ*_*f*_, i.e.,

$$\text{CCW}\overset{\lambda_{f}}{\underset{\mu_{f}}{⇌}}\text{CW}.$$

These rates have been estimated using experimental data [21, 22],

$$\begin{array}{c} \lambda_{f}=a_{1}\exp\left(b_{1}Y_{p}\right), \end{array}$$ (7)

$$\begin{array}{c} \mu_{f}=a_{2}\exp(-{\left(b_{2}-Y_{p}\right)}^{4}/c), \end{array}$$ (8)

where *a*_1_, *b*_1_, *a*_2_, *b*_2_ and *c* are constants specified in Table 2. The fitting is replotted in S1 Text for readers’ convenience. We note that given *z* and *m* for each cell, *Y*_*p*_ = *Y*_*p*_(*m*, *z*) can be solved from (5) and used to determine λ_*f*_ and *μ*_*f*_.

**Table 2:** Parameters for cell movement and cell growth.

Param.	Description	Value
*s*_0_	cell speed	0.02mm · s^−1^
*a*_1_	coefficient in Eq (11)	0.0174001 *s*^−1^
*b*_1_	coefficient in Eq (11)	1.32887 *μM*^−1^
*a*_2_	coefficient in Eq (12)	12.0809 *s*^−1^
*b*_2_	coefficient in Eq (12)	-5.83762 *μM*
*c*	coefficient in Eq (12)	2892.12
*n*_*f*_	total flagella number	8
*w*	minimum number of CCW flagella needed to run	6
*r*	cell growth rate	3.85 × 10^−4^s^−1^

We assume that each cell has *n*_*f*_ flagella that rotate independently. If at least *w* flagella rotate CCW simultaneously then the cell runs forward; otherwise it tumbles in place. In reality different flagella may interact with each other through the surrounding fluid, but we ignore this effect for simplicity.

The probability of having exactly *i* flagella rotating CCW is given by

$$\begin{array}{c} P_{CCW}^{i}=\left(\begin{array}{c} n_{f}\\ i \end{array}\right){\left(\frac{\mu_{f}}{\lambda_{f}+\mu_{f}}\right)}^{i}{\left(\frac{\lambda_{f}}{\lambda_{f}+\mu_{f}}\right)}^{n_{f}-i}. \end{array}$$ (9)

The probability for the cell to be in the run and tumble states are given by

$$\begin{array}{c} P_{run}=\sum_{i=w}^{n_{f}}P_{CCW}^{i},\;P_{tumble}=1-P_{run}. \end{array}$$ (10)

The probability for two flagella switching rotation simultaneously is very small; therefore the switch from run to tumble primarily occurs when the cell has exactly *w* flagella rotating CCW and one of them switches to CW direction. Based on these observations, λ can be estimated as

$$\begin{array}{c} \lambda \left(m,z\right)=w\lambda_{f}\cdot \frac{P_{CCW}^{w}}{P_{run}}. \end{array}$$ (11)

Similarly, a cell switches from tumble to run primarily when there are exactly *w* − 1 flagella rotating CCW at that moment and one of the rest switches to CCW. This argument leads to

$$\begin{array}{c} \mu \left(m,z\right)=\left(n_{f}-w+1\right)\mu_{f}\cdot \frac{P_{CCW}^{w-1}}{P_{tumble}}. \end{array}$$ (12)

Using *n*_*f*_ = 8, *w* = 6 and total CheZ concentration 1.23*μM*, we obtain λ = 0.594*s*^−1^ and *μ* = 6.1143*s*^−1^ at basal CheYp level, which is consistent with experimental data for wild-type cells (See S1 Text).

#### Cell growth

We assume that the growth rate of the cells is a linear function of the local nutrient concentration *n*(**x**, *t*), i.e.,

$$\begin{array}{c} k_{V}\left(\text{x},t\right)=rn\left(\text{x},t\right). \end{array}$$ (13)

Furthermore we model cell proliferation as a Poisson process, i.e., during the time interval [*t*, *t* + *dt*), the probability for a cell to divide into two daughter cells is *k*_*V*_(**x**, *t*)*dt*. An alternative approach is to introduce a cell cycle variable for each cell and divide it when it doubles in size [23]. We tested both approaches numerically and found no visible difference. For this reason, we used the Poisson process approach in this paper for the ease of mathematical analysis in S2 Text.

We assume that the cell doubling time is approximately 30 minutes at maximum nutrient level and regard *n*(**x**, *t*) as the nutrient concentration normalized by its initial value. Therefore we have *r* = *ln*2/(30min) ≈ 3.85 × 10^−4^s^−1^ (Table 2).

#### AHL and nutrient dynamics

Denote the total number of bacteria at time *t* as *n*_*b*_. We assume that AHL is secreted by each cell with a constant rate *α*_*d*_ and degrades naturally. We further assume that the nutrient is consumed by each cell at a rate proportional to the nutrient concentration. Based on these assumptions, we have

$$\begin{array}{c} \begin{array}{cc} & \partial_{t}h\left(\text{x},t\right)=D_{h}\Delta h\left(\text{x},t\right)+\alpha_{d}\sum_{i=1}^{n_{b}}\delta \left(\text{x}-{\text{x}}_{i}\right)-\beta h\left(\text{x},t\right),\\ & \partial_{t}n\left(\text{x},t\right)=D_{n}\Delta n\left(\text{x},t\right)-\gamma_{d}\sum_{i=1}^{n_{b}}\delta \left(\text{x}-{\text{x}}_{i}\right)n\left(\text{x},t\right). \end{array} \end{array}$$ (14)

The parameters for AHL and nutrient dynamics are listed in Table 3. Since different experimental conditions can lead to different parameter values which were not reported in [12], they effect the patterns and we will explore the parameters more later on.

**Table 3:** Parameters for AHL and the nutrient.

Param.	Description	Value
*D*_*h*_	diffusion coefficient of *AHL*	5 × 10^−4^mm^2^ · s^−1^
*D*_*n*_	diffusion coefficient of the nutrient	7.7 × 10^−4^mm^2^ · s^−1^
*α*_*d*_	production rate of AHL per cell	10^−6^ *s*^−1^
*β*	degradation rate of AHL	10^−3^ *s*^−1^
*γ*_*d*_	consumption rate of nutrient per cell	1.155 × 10^−6^ *s*^−1^
*n*_0_	Initial concentration of nutrient (non-dimensional)	1

### PDE model

To reduce computational cost, we derived a PDE model from the hybrid model using moment closure methods and asymptotic analysis. Let *p*(***x***, ***v***, *m*, *z*, *t*) be the density of cells at position ***x***, with velocity *v*, internal states *m* and *z*, and at time *t*. Let *p*_0_(***x***, *m*, *z*, *t*) be the density of cells resting at position ***x*** with internal states *m* and *z*. According to the hybrid model we have

$$\begin{array}{c} \begin{array}{cc} & \partial_{t}p+v\cdot \nabla_{x}p+\partial_{z}\left(g\left(z,h\right)p\right)+\partial_{m}\left(f\left(m,z\right)p\right)=Q(p,p_{0}),\\ & \partial_{t}p_{0}+\partial_{z}\left(g\left(z,h\right)p_{0}\right)+\partial_{m}\left(f\left(m,z\right)p_{0}\right)=Q_{0}(p,p_{0}). \end{array} \end{array}$$ (15)

Here *g*(*z*, *h*) and *f*(*m*, *z*) are the right-hand sides of Eqs (1) and (2), and

$$\begin{array}{c} \begin{array}{cc} & Q\left(p,p_{0}\right)=-\lambda \left(m,z\right)p+\mu \left(m,z\right)p_{0}/\left|V\right|+rnp,\\ & Q_{0}\left(p,p_{0}\right)=\lambda \left(m,z\right)\int_{V}pdv-\mu \left(m,z\right)p_{0}+rnp_{0}, \end{array} \end{array}$$ (16)

where $V=s_{0}\partial B_{0}^{1}$, λ(*m*, *z*), *μ*(*m*, *z*) are given by Eqs (11) and (12), and *n* = *n*(**x**, *t*) is the local nutrient concentration. The first two terms in *Q*(*p*, *p*_0_) and *Q*_0_(*p*, *p*_0_) represent the density change due to velocity jumps and the third terms are due to cell growth.

Let *ρ*^*z*^(**x**, *z*, *t*) be the density of cells at position **x** with internal state *z*, then

$$\rho^{z}=\int_{R}(p_{0}+\int_{V}pdv)dm.$$

We derived the following approximating equation for *ρ*^*z*^(**x**, *z*, *t*) from (15) and (16) (see S2 Text),

$$\begin{array}{c} \begin{array}{cc} & \partial_{t}\rho^{z}=\nabla_{x}\cdot (D\left(z\right)\nabla_{x}\rho^{z})-\partial_{z}\left(g\left(z,h\left(x,t\right)\right)\rho^{z}\right)+rn\rho^{z}. \end{array} \end{array}$$ (17)

Here *h* is the AHL concentration and

$$\begin{array}{c} D\left(z\right)=\frac{s_{0}^{2}\mu_{0}\left(z\right)}{d\lambda_{0}\left(z\right)\left[\mu_{0}\left(z\right)+\lambda_{0}\left(z\right)\right]}, \end{array}$$ (18)

where *d* is the space dimension, and λ_0_(*z*) and *μ*_0_(*z*) are the switching frequencies when *m* equals its quasi-steady state. We note that the intracellular chemotactic signaling enters into Eq (17) through the quasi-steady state of *m* only. This is because the methylation time scale is much smaller than the time scale for the change of *z*.

The derivation was based on time scale separation of the intrinsic biological processes: the time scale for chemotactic signaling is seconds to minutes, the time scale for CheZ dynamics is tens of minutes, and the time scale for the stripe formation is several hours. The derivation involves moment closure methods and asymptotic analysis, similar to our previous works [15, 17, 18, 24].

The PDE model is formed by coupling Eq (17) with the continuous version of (14), namely,

$$\begin{array}{c} \begin{array}{cc} & \partial_{t}h\left(x,t\right)=D_{h}\Delta h\left(x,t\right)+\alpha \rho \left(x,t\right)-\beta h\left(x,t\right),\\ & \partial_{t}n\left(x,t\right)=D_{n}\Delta n\left(x,t\right)-\gamma \rho \left(x,t\right)n\left(x,t\right), \end{array} \end{array}$$ (19)

where

$$\rho \left(x,t\right)=\int \rho^{z}\left(x,z,t\right)\;dz.$$

The parameters of the PDE model are fully determined by those of the hybrid model. In our simulations we choose the cell density scale to be *ρ_s_* = 1000cells · mm^−1^. As a consequence, *α* and *γ* can be calculated as *α* = *α*_*d*_ *ρ*_*s*_ and *γ* = *γ*_*d*_ *ρ*_*s*_.

## Results

### How does single cell dynamics depend on total CheZ

We first investigated how intracellular signaling and cell movement depend on the total concentration of CheZ protein (denoted as *Z*_*t*_ in this section).

#### Dependence of intracellular signaling on total CheZ

**Fig 3:**
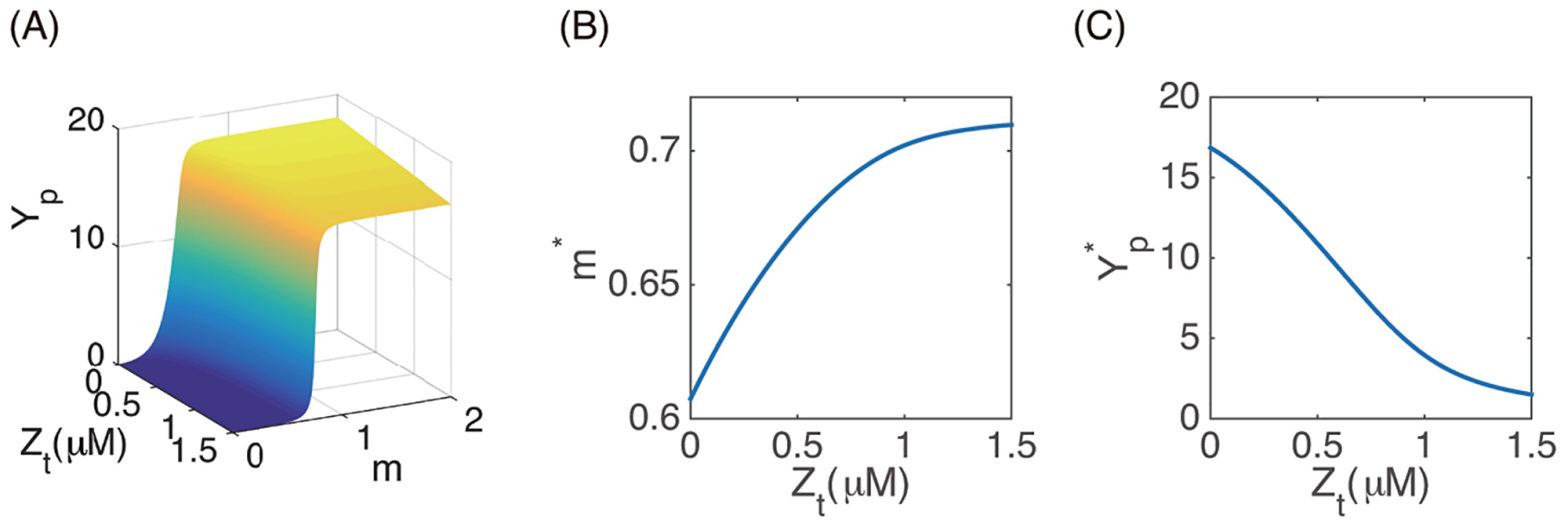
Dependence of intracellular signaling on total CheZ. (A) *Y*_*p*_ as a function of *Z*_*t*_ and *m*. (B) the stationary methylation level m* as a function of *Z*_*t*_; (C) the stationary CheYp concentration $Y_{p}^{*}$ given different *Z*_*t*_. Parameter values are the same as in Table 1.

CheYp is the intracellular protein that binds to a cell’s flagellar motor and changes its rotation (Fig 1B). CheYp concentration (*Y*_*p*_) depends on total CheZ (*Z*_*t*_) and the methylation state of the receptors (*m*) in a ultra-sensitive manner. The relation was solved from (5) and (6) and plotted in Fig 3A. Total CheZ in a cell changes on a much slower time scale (tens of minutes) than the methylation and demethylation of the cell receptors (several seconds to minutes). As a result, in the absence of external receptor-binding signals, *m* and *Y*_*p*_ are close to their steady states m* and $Y_{p}^{*}$. Figs 3B and 2C plot m* and $Y_{p}^{*}$ obtained using the ODE model (1)–(6). We note that these variables vary significantly with *Z*_*t*_. This implies that cells can demonstrate chemotactic-like behavior if *Z*_*t*_ changes along their trajectories.

#### Dependence of cell movement on total CheZ

**Fig 4:**
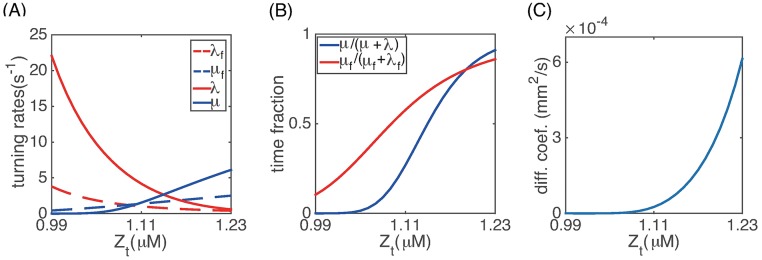
(A) Dependence of the turning rates on *Z_t_*; (B) Mean time fraction of CCW rotation and cell running as a function of *Z_t_*. (C) Effective cell diffusion coefficient calculated using Eq (18).

Assuming *m* = m*, we calculated the turning rates for a single flagellum (λ_*f*_, *μ*_*f*_) as well as for the whole cell (λ, *μ*) as a function of *Z*_*t*_. Fig 4A shows that λ_*f*_ and λ decrease with *Z*_*t*_, while *μ*_*f*_ and *μ* increase with *Z*_*t*_. The mean time fraction that a single flagella spends in the CCW rotation is given by *μ*_*f*_/(λ_*f*_ + *μ*_*f*_), and the mean time fraction that a cell spend in the running state is *μ*/(λ + *μ*). Fig 4B shows that these quantities are very sensitive to *Z*_*t*_ and decrease significantly if *Z*_*t*_ is reduced: cells spend 90% of their time running if *Z*_*t*_ = *Z*_*w*_ ≈ 1.23*μM* but only 25% if *Z*_*t*_ is reduced to 1.11 *μM*. Moreover, the velocity jump of a cell is more sensitive to *Z*_*t*_ than the rotation direction change of a single flagellum due to the cooperative behavior of different flagella. These results reconfirm the significance of the tumbling state in periodic stripe formation.

Fig 4C plots the effective diffusion coefficient for cell movement in 1D calculated using Eq (18). Experimental measurements in [12] showed that the cell diffusion rate decreases sharply with increasing cell density. Our calculation is consistent with experimental data and gives an explanation of this relation at the molecular level: high cell density is associated with high AHL concentration, which shuts off the production of CheZ inside the cells. This in turn causes *Z*_*t*_ to decrease and cells tumble in place extensively.

### Quantitative agreement between hybrid and PDE models in 1D

If cells are initially seeded on a horizontal line in an agar plate, they will grow, spread out laterally and form straight stripes of equal spacing (see Fig. S4 of [12]). Motivated by these experiments, we first investigated the population pattern formation on a 1D domain [−*L*, *L*], representing a cross-section of the stripe patterns, using the hybrid model and the PDE model. Simulations suggest that both models predict the same spatial-temporal population dynamics for the engineered stripe-forming mutants as well as wild-type cells as in experiments. Moreover, the derived PDE model agrees with the hybrid model quantitatively in biologically-relevant parameter regimes.

To mimic the experimental setup, we assumed that all cells initially cluster near the center (*x* = 0) with internal states at equilibrium, i.e., *z* = *Z*_*w*_ and *m* = *m*_0_. Specifically, for the hybrid model, we randomly put 500 cells in the domain according to the distribution

$$\begin{array}{c} P\left(x\right)=\frac{1}{\sigma \sqrt{2\pi }}\text{exp}\left(-\frac{x^{2}}{2\sigma^{2}}\right) \end{array}$$

with *σ* = 2mm at *t* = 0. Correspondingly, for the PDE model, we took

$$\rho^{z}\left(x,z,0\right)=\rho_{0}P\left(x\right)\delta \left(z-Z_{w}\right),$$

where *ρ*_0_ = 500cells · mm^−1^/*ρ*_s_ = 0.5 (*ρ*_s_ = 1000cells · mm^−1^ is the cell density scale). For both models, we took the initial nutrient concentration to be a constant everywhere and assumed that there was no AHL added in the domain, i.e.,

n(x,0)=1,h(x,0)=0.

We used no-flux boundary conditions throughout the paper. For AHL and nutrient concentrations, we imposed ∇*h* ⋅ **n** = ∇*n* ⋅ **n** = 0 at the boundary of the spatial domain, where **n** is the outward normal vector. For individual cell movement, we assumed that once a cell reaches the boundary, it bounces back with its velocity reflected by the boundary. In 1D, the cell direction simply reverses. For Eq (17), we chose the computational range *z* ∈ [*z*_*min*_, *z*_*max*_] to be large enough to include all possible CheZ concentrations such that *ρ*^*z*^(**x**, *z*_*min*_, *t*) = *ρ*^*z*^(**x**, *z*_*max*_, *t*) = 0. In the **x** direction, we imposed that ∇*ρ*^*z*^ ⋅ **n** = 0 for all *z*.

**Fig 5:**
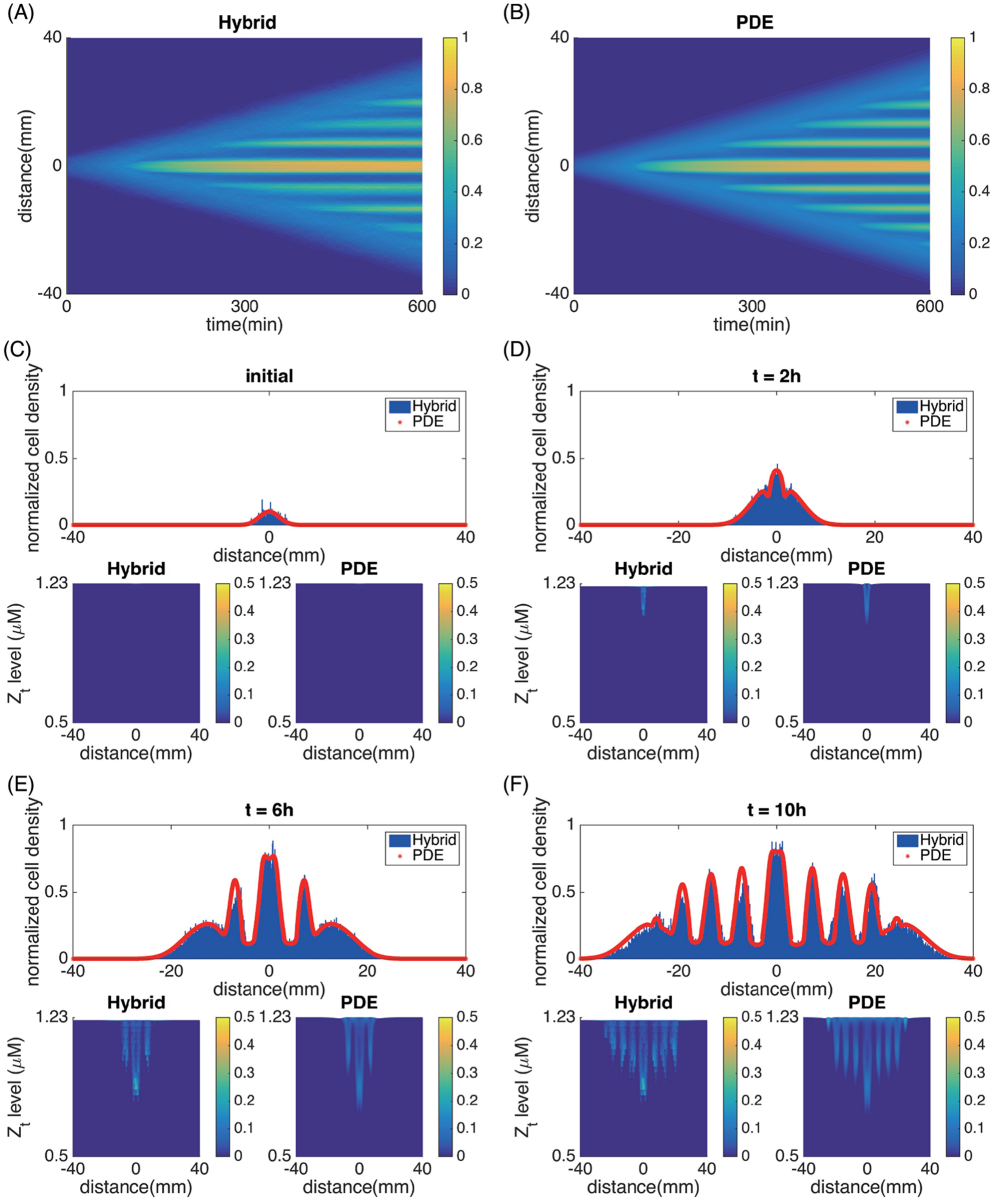
Sequential stripe formation in 1D for engineered mutants. (A) spatial-temporal evolution of the cell density recovered from averages of 6 realizations of the hybrid model. (B) the evolution of the cell density from the PDE model. (C)—(F): spatial plots of the cell density and *Z*_*t*_ distribution at *t* = 0h, *t* = 2h, *t* = 6h and *t* = 10h, respectively. Top: the normalized cell density *ρ*(*x*, *t*). Blue bars are obtained from histograms using the hybrid model, averaged over 6 realizations. Red Lines are calculated from the PDE model. Bottom left: distribution of *ρ*^*z*^(*x*, *z*, *t*) in space and time from the hybrid model. Bottom right: *ρ*^*z*^(*x*, *z*, *t*) from the PDE model. The parameters used here are the same as in Tables 1–3.

We first simulated the cell population dynamics for the stripe-forming mutants with parameters specified in Tables 1–3. Fig 5 presents the time course data of the cell density as well as the distribution of the internal variable *Z*_*t*_. Panels A and B are the heat maps of the cell density as a function of space and time. The normalized cell density for the PDE model was obtained by integrating *ρ*^*z*^ over *z*. The normalized cell density for the hybrid model was calculated using histograms of the cell positions. Panels C-F present the detailed comparisons of the normalized cell density in space (top) as well as the *Z*_*t*_ distribution (bottom) given by the two approaches at different time points. The *Z*_*t*_ distribution was obtained by normalizing the cell number in each rectangular grid with size 0.1mm × 0.03*μ*M by 100 cells. In these simulations, the AHL concentration *h* also shows the same stripe pattern as the cell density *ρ*, with peaks and valleys coinsiding with those of *ρ*; while the nutrient forms a wave front at the colony front, increasing from 0 to the initial normalized state.

Fig 5 shows that the total cell number grows significantly as cells divide. As the colony grows, it propagates outward continuously with a more or less constant front speed. Meanwhile cells produce AHL continuously. As extracellular AHL concentration becomes high and reaches the threshold *h*_0_ locally, the intracellular *Z*_*t*_ at these locations start to drop. As a result, cells at these locations spend more time in the tumbling stage and become less mobile. In contrast, cells in nearby regions with low AHL move more persistently until they migrate into a high AHL region. The existence of high and low mobility regions leads to the sequential establishment of high-density stripes behind the colony front, similar to experiments.

**Fig 6:**
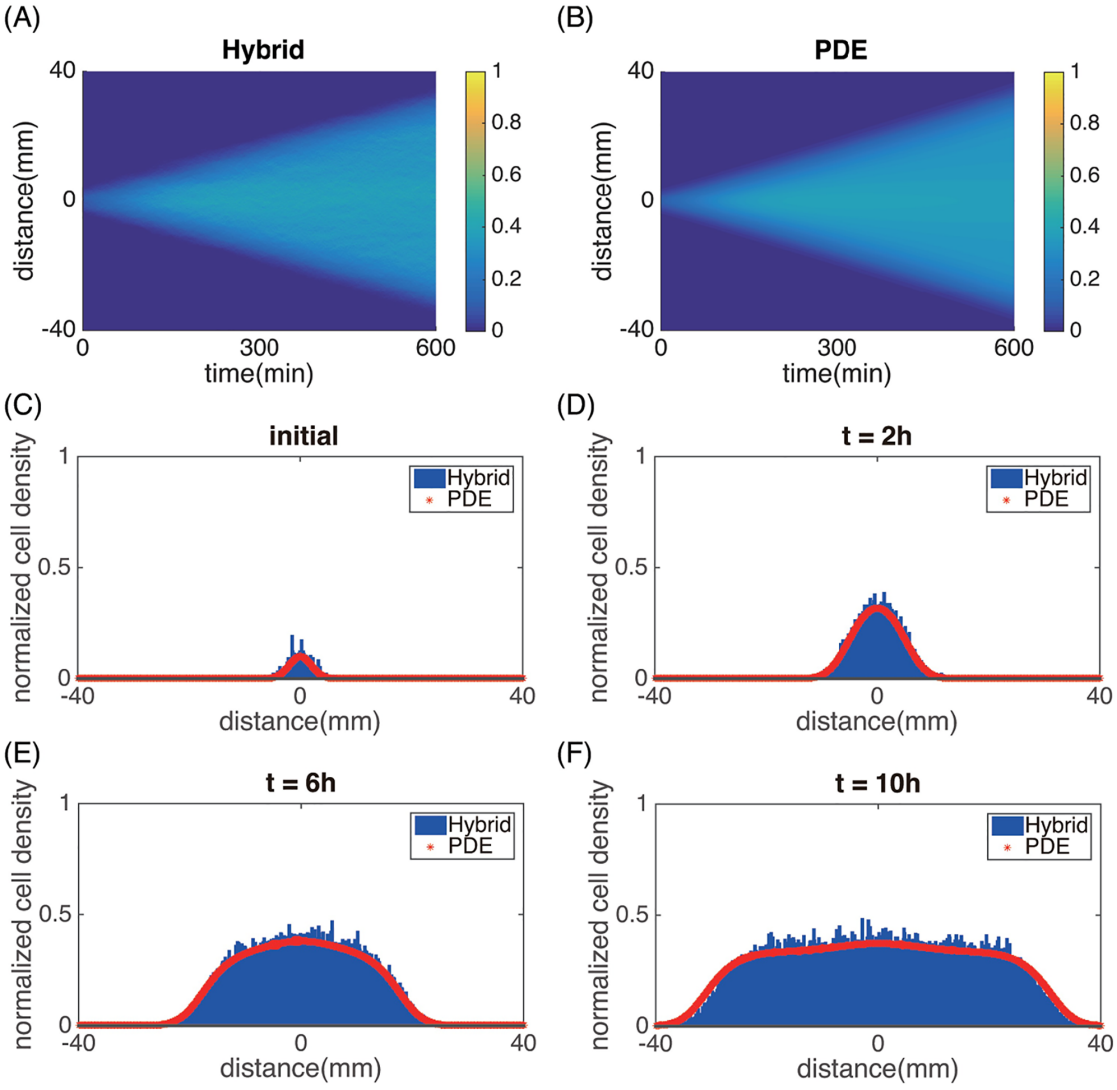
Uniform colony expansion for wild-type cells. (A) spatial-temporal evolution of the cell density recovered from averages of 6 realizations of the hybrid model. (B) The evolution of the cell density from the PDE model. (C)—(F) Spatial plots of the cell density at *t* = 0h, *t* = 2h, *t* = 6h and *t* = 10h, respectively. Blue bars are obtained from histograms using the hybrid model averaged over 6 realizations. Red Lines are calculated from the PDE model. In these simulations we used *α*_*d*_ = *α* = 0. Other parameters are the same as in Tables 1–3.

As a comparison, we then simulated the population dynamics for wild-type cells that do not secrete AHL, i.e., *α*_*d*_ = *α* = 0 (Fig 6). In this case, cells grow, consume nutrients, and the colony propagates outward with a constant wave front speed. However, stripes do not appear behind the colony front.

Figs 5 and 6 suggest quantitative agreement between the hybrid model and the PDE model. This justifies using the PDE model for further investigations to save computational cost. We also note that the colony front expansion speed for both the engineered mutant and wild-type are identical. This is because the front speed is primarily determined by the growth and motility of cells at the colony front, where AHL does not reach the threshold *h*_0_ required for quorum-sensing. Hence, cells therein have the wild-type phenotype for both cases.

### Concentric stripe formation in 2D predicted by the PDE model

#### Stripe patterns on a plate

Using the PDE model, we next investigated how the concentric stripe pattern in Fig 1 forms from a single inoculant in the center of the petri dish. Assuming radial symmetry, the PDE model takes the following form

$$\begin{array}{l} \partial_{t}\rho^{z}=\frac{1}{\xi }\partial_{\xi }\left(D\left(z\right)\xi \partial_{\xi }\rho^{z}\right)-\partial_{z}\left(g\left(z,h\left(\xi ,t\right)\right)\rho^{z}\right)+rn\rho^{z},\\ \partial_{t}h\left(\xi ,t\right)=\frac{D_{h}}{\xi }\partial_{\xi }\left(\xi \partial_{\xi }h\left(\xi ,t\right)\right)+\alpha \rho \left(\xi ,t\right)-\beta h\left(\xi ,t\right),\\ \partial_{t}n\left(\xi ,t\right)=\frac{D_{n}}{\xi }\partial_{\xi }\left(\xi \partial_{\xi }n\left(\xi ,t\right)\right)-\gamma \rho \left(\xi ,t\right)n\left(\xi ,t\right), \end{array}$$ (20)

where *ξ* ∈ (0, *R*] is the polar coordinate. We imposed the following boundary conditions

$$\begin{array}{c} \begin{array}{cc} & \partial_{\xi }\rho^{z}\left(0,z,t\right)=\partial_{\xi }\rho^{z}\left(R,z,t\right)=0,\;\rho^{z}\left(\xi ,0,t\right)=\rho^{z}\left(\xi ,Z_{\max},t\right)=0,\\ & \partial_{\xi }h\left(0,t\right)=\partial_{\xi }h\left(R,t\right)=0,\;\partial_{\xi }n\left(0,t\right)=\partial_{\xi }n\left(R,t\right)=0 \end{array} \end{array}$$ (21)

The initial conditions mimics the initial conditions in the experiments [12]

$$\begin{array}{c} \begin{array}{ccc} \rho^{z}\left(\xi ,z,0\right)=\frac{1}{4\sqrt{2\pi }}\text{exp}\left(-\frac{\xi^{2}}{8}\right)\delta \left(z-Z_{w}\right), & h\left(\xi ,0\right)=0, & n\left(\xi ,0\right)=1. \end{array} \end{array}$$ (22)

**Fig 7:**
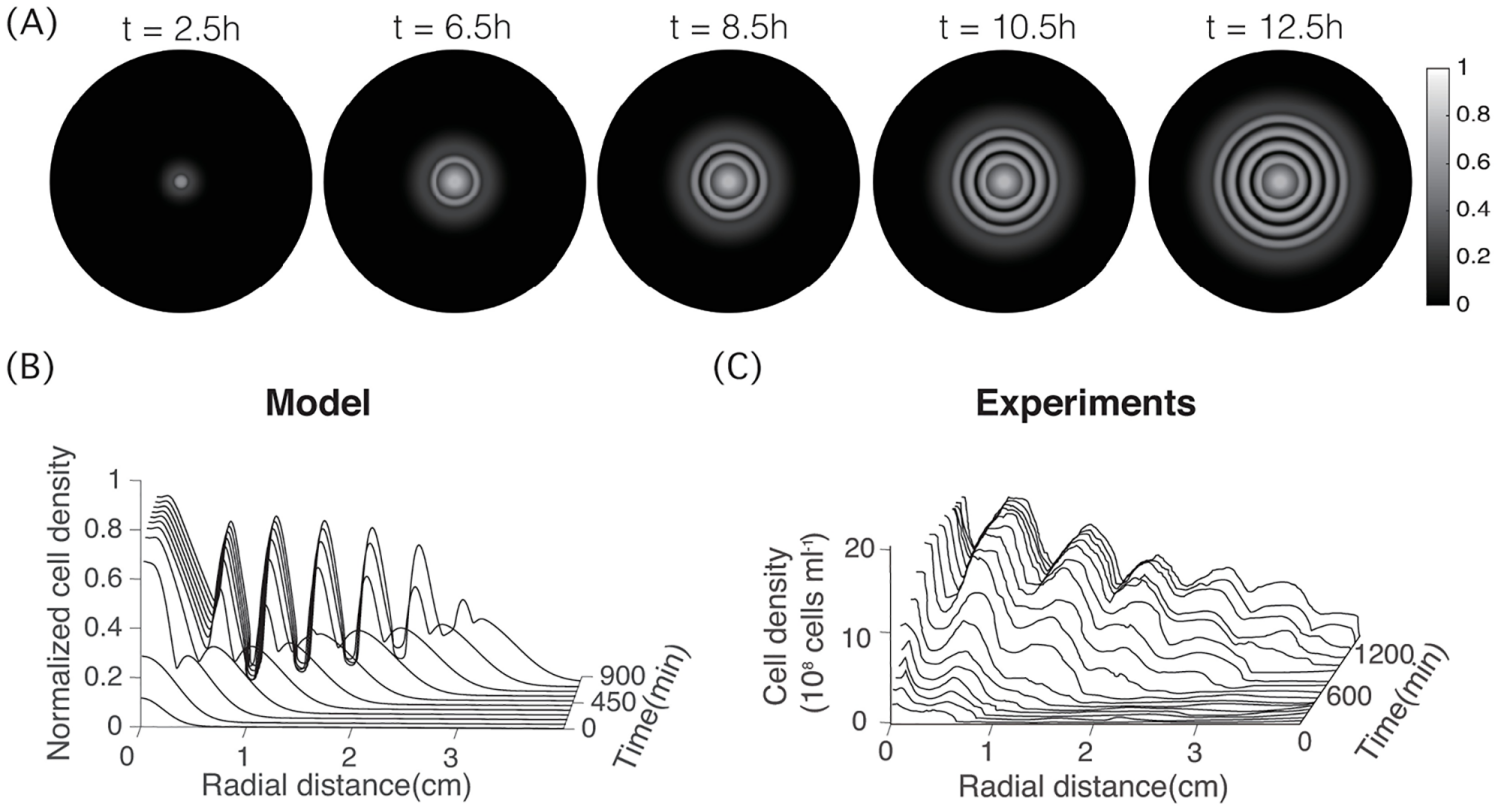
Concentric stripe patterns in 2D predicted by the PDE model. (A) spatial density plots at *t* = 2.5h, *t* = 6.5h, *t* = 8.5h, *t* = 10.5h and *t* = 12.5h. (B) Dynamics of the cell density in the radial direction predicted by the PDE model. (C) Dynamics of the cell density in the radial direction measured in experiments. (C) Reproduced from Fig. S3 in SOM of Liu et al, Science, Vol 334, 238–241, 2011 [12]. Parameters used here are either directly taken from Tables 1–3 or calculated using the conversion formulas.

According to the experiments in [12], the initial cell colony expanded for several hours before its density reaches the threshold to form a stripe. Each stripe is composed of a high density part and a low density part with the average wavelength approximately 0.5cm. The average time to form one stripe is around 200mins (see Fig 1). Simulations of our PDE model with baseline parameters predict spatiotemporal dynamics that agrees with experiments semi-quantitatively (Fig 7).

#### Effect of CheZ inhibitor

In [12], the expression of cheZ gene was varied using an aTc-inducible module. As aTc level increases, the *CheZ* mRNA level decreases gradually. At high aTc level, the engineered *E. coli* colonies do not form the spatial stripe patterns as they grow. Motivated by these experiments, we investigated how the population pattern formation depends on the synthesis rate of CheZ protein. Specifically, we varied the parameter *Z*_*w*_ in Eq (1), which is the steady state of CheZ concentration in the absence of quorum-sensing effect.

**Fig 8:**
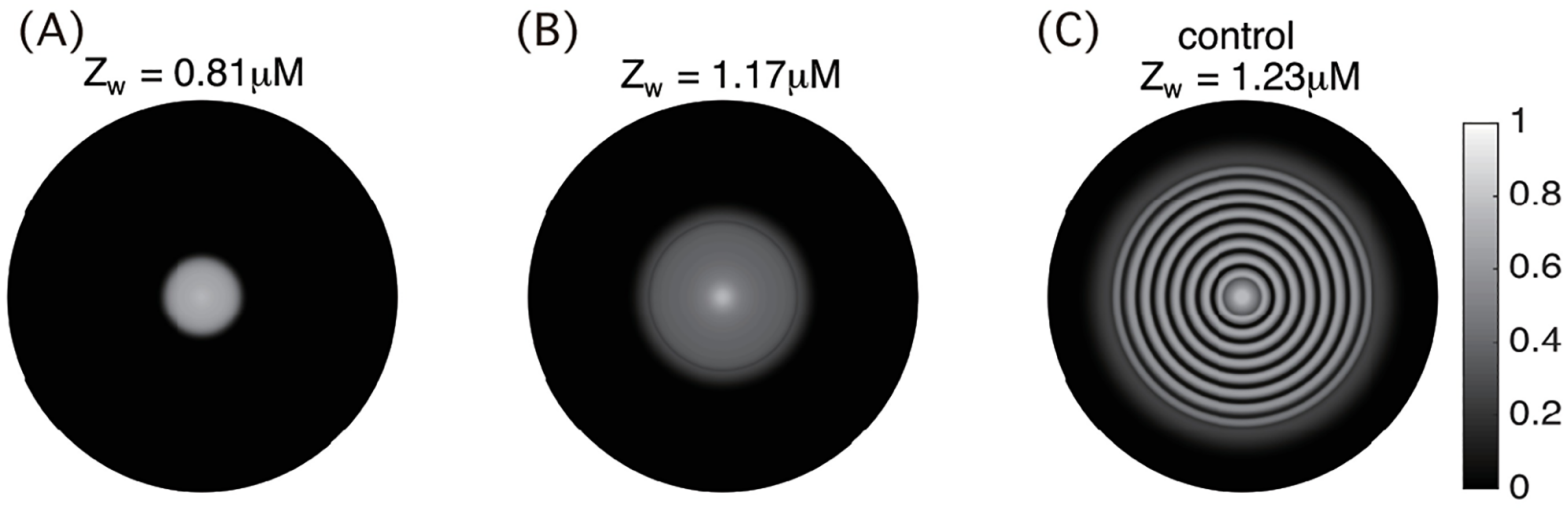
CheZ inhibitor disrupts the stripe pattern formation. The effect of CheZ inhibitor aTc is modeled by a reduction of total CheZ protein level *Z*_*w*_. (A)–(C): cell density predicted by the PDE model at *t* = 20h with *Z*_*w*_ = 0.81*μM*, *Z*_*w*_ = 1.17*μM* and *Z*_*w*_ = 1.23*μM* (no inhibitor), respectively. Other parameters are the same as in Fig 7.

Fig 8 shows that as *Z*_*w*_ decreases, the colony expansion rate decreases and the stripe patterns disappear, consistent with experimental data. In our simulations, small changes in *Z*_*w*_ induce large variances in the colony expansion rate. This is because the effective diffusion coefficient of the cells is very sensitive to the total CheZ level (Fig 4C). The experiments in [12] measured the correlation between *CheZ* mRNA level and the pattern formation. We note that the magnitudes of changes in CheZ protein and *CheZ* mRNA can be different in these experiments.

#### Effect of CheR CheB mutation

In [12], a secondary mutation was introduced to the engineered cell line by knocking out the *CheR* and *CheB* genes. The purpose was to investigate whether canonical chemotaxis induced by external ligands is a critical factor in the stripe pattern formation. Cells can still swim and tumble, but cannot adapt to chemotactic signals because the methylation level of the receptors are not modified. Experiments showed that these mutants can still form sequential stripe patterns, but there was little discussion on how the pattern differs from those formed by non-mutants (Fig S2(J) in [12]). The conclusion was that canonical chemotaxis is not necessary for the spatial pattern formation.

**Fig 9:**
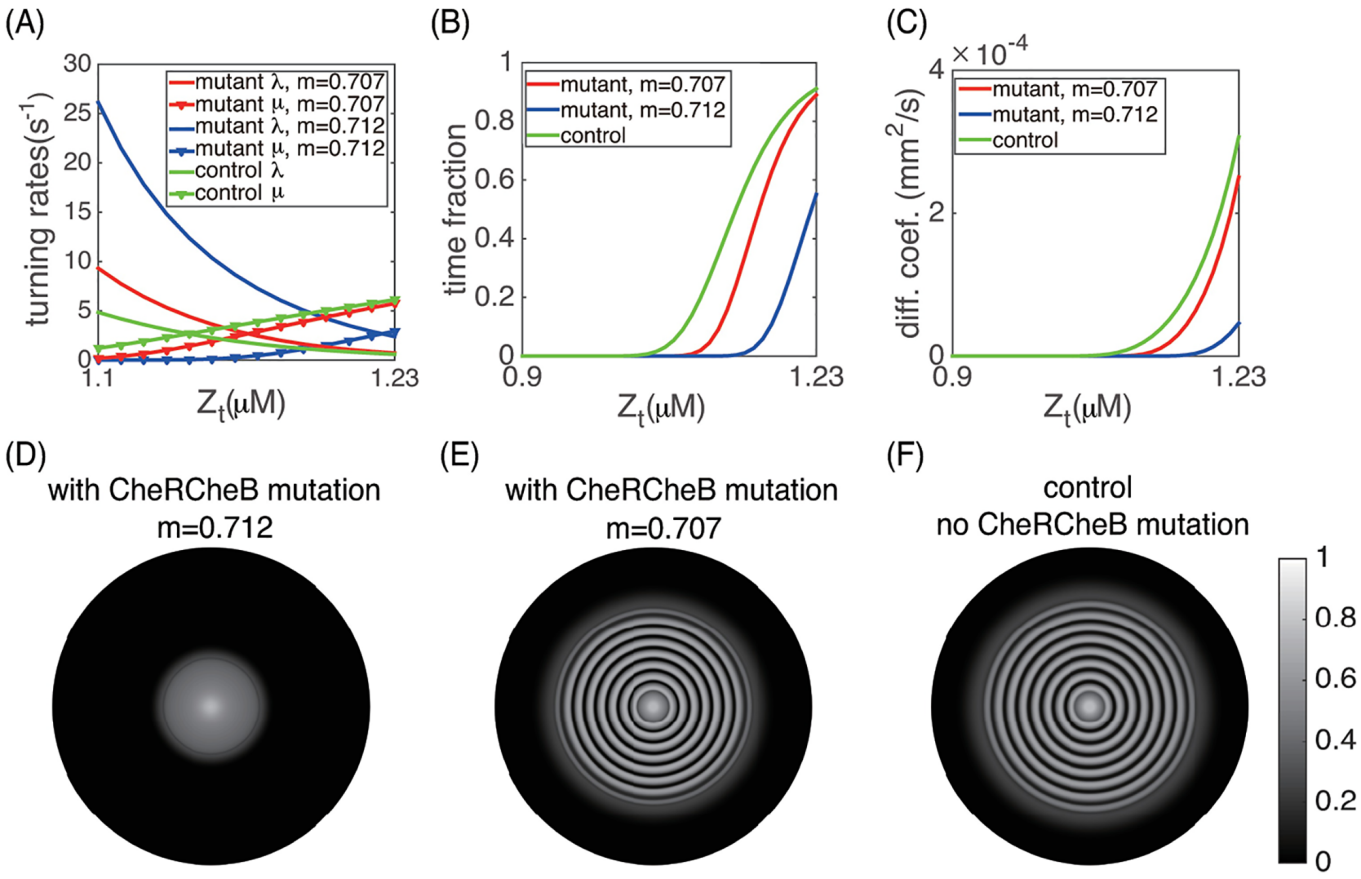
The effect of *CheR*^−1^ *CheB*^−1^ double mutation. (A) Dependence of cell turning rates on *Z*_*t*_. (B) Mean time fraction that cell spends running as a function of *Z*_*t*_, denoted as $\tilde{\mu }$. (C) Effective diffusion coefficients (Eq (18) for different *Z*_*t*_. The methylation level *m* is kept constant for the mutant cells. (D) and (E): cell density plots at *t* = 20h for mutant cells with *m* = 0.712 and *m* = 0.707 respectively. (F) cell density plot for the engineered cells without secondary mutation as a comparison. Other parameters are the same as in Fig 7.

We investigated this aspect numerically using the PDE model (Fig 9). Knocking out *CheR*, *CheB* indicates that *R*, *B*_*t*_ and *B*_*p*_ are all zeros in (2)–(6). Thus according to our model, *m* remains a constant which depends on the initial methylation level of the cells. Due to mutation, the cell turning rates and the effective cell diffusion coefficients are modified (A, B, C). The effective cell diffusion coefficient is very sensitive to the methylation level *m* (C). This is because the dependence of *Y*_*p*_ on the methylation level *m* has a sharp transition, similar to Fig 3A. Simulations of the corresponding PDE model showed that the colony expansion rate and the interior spatial pattern are both very sensitive to the initial methylation level of the cells: small changes in *m* can lead to big differences in the population dynamics (D, E). In experiments, the methylation level of each bacterium also fluctuates due to internal noise. These considerations suggest that it is not justified to use this cell line to draw conclusions on whether canonical chemotaxis is involved in the pattern formation process.

### How does the stripe formation depend on cell-level parameters

We use three important features to characterize the spatial-temporal pattern: the colony front propagation speed, the wavelength of the spatial stripes and the internal structure within a spatial period. We investigated how these features depend on intracellular dynamics, cell movement and cell growth.

**Fig 10:**
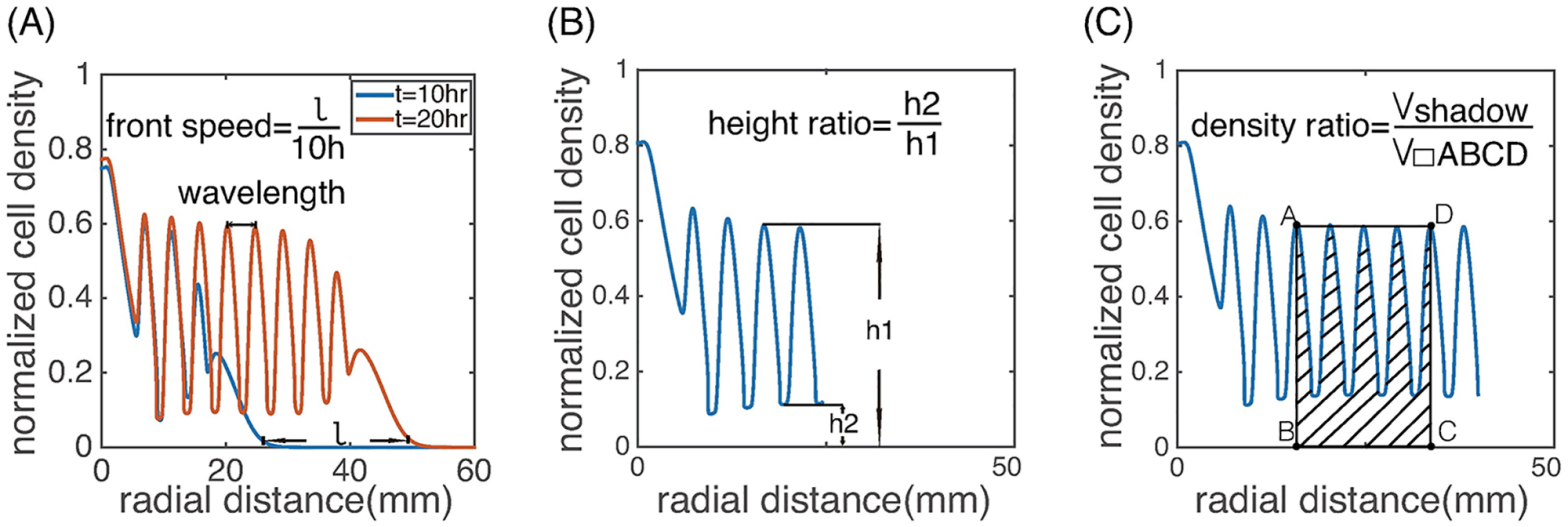
Schematic illustration of the front speed, wavelength, height ratio and density ratio.

We calculated the front speed as the average speed between *t* = 10 hr and 20 hr and the wavelength as the average distance between the maximum densities of two successive high density stripes (Fig 10A). To characterize the internal structure of the stripes, we defined the height ratio and the density ratio of the stripes (Fig 9B and 9C). The height ratio is the minimum density (*h*_2_) divided by the maximum density (*h*_1_) within a stripe. The density ratio is the volume of the shaded region over the region defined by the rectangle ABCD, factoring in the radially symmetric profile of the solution, i.e., $\int_{B}^{C}\xi \rho \left(\xi ,t\right)/h_{1}d\xi$. The height ratio measures the fluctuations of the cell density in the spatial pattern; while the density ratio quantifies the area fraction of the high-density regions when *h*_2_ is small.

#### Dependence on cell speed and cell doubling time

**Fig 11:**
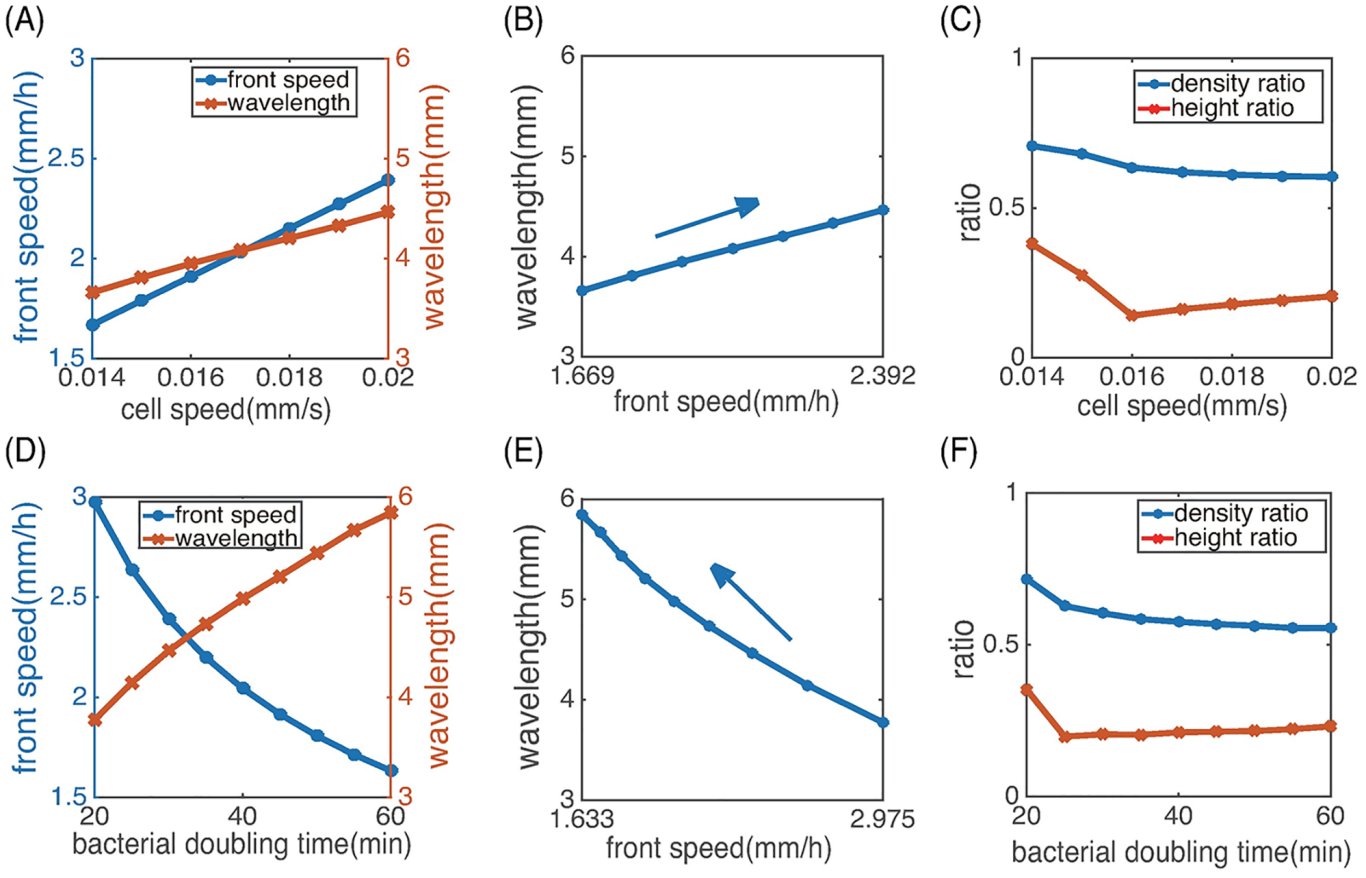
Dependence on individual cell speed and cell doubling time. (A), (D) Dependence of the front speed and the pattern wavelength on the cell speed and doubling time. (B), (E) The linear dependence between the pattern wavelength and the front speed. The cell speed or doubling time increases along the directions of the arrows. (C), (F) The height ratio and the density ratio as functions of the cell speed and doubling time. All other parameters are the same as in Fig 7.

We investigated how the stripe pattern depends on the individual cell speed (*s*_0_) and the cell doubling time (log2/*r*) by varying the parameters *s*_0_ and *r* in (18) and (20). Simulations of the PDE model show that as the cell speed increases, both the colony front speed and the pattern wavelength increase linearly (Fig 11A and 11B). If individual cells move faster near the colony front, the effective diffusion rate of the population becomes larger and thus the colony spreads out faster. Indeed, our simulation shows that the colony front speed is roughly proportional to the cell speed. As the cell doubling time increases, the front speed decreases, but the pattern wavelength increases, reflecting the slower growth of the total population size (Fig 11D and 11E). Our model predicts a linear dependence of the front speed and the pattern wavelength as one varies the cell speed (Fig 11B) or the cell doubling time (Fig 11E). The height ratio and the density ratio only decrease slightly with the cell speed and doubling time (Fig 11C and 11F).

#### Dependence on the CheZ turnover rate

In our models, we associated the turnover rate of CheZ protein with the cell growth rate *k*_*V*_, see Eq (1). We next investigated the effect of a slower or faster CheZ turnover rate on the spatial patterning. To do that, we introduced a nondimensional parameter *κ* preceding the *z*-flux term in Eq (17), i.e.,

$$\begin{array}{c} \partial_{t}\rho^{z}=\frac{1}{\xi }\partial_{\xi }\left(D\left(z\right)\xi \partial_{\xi }\rho^{z}\right)-\kappa \partial_{z}\left(g\left(z,h\left(\xi ,t\right)\right)\rho^{z}\right)+r\rho^{z}n. \end{array}$$ (23)

Mathematically speaking, *κ* parameterizes the convective speed in *z*, which corresponds to the response speed of intracellular CheZ to the external signal AHL: *κ* = 1 represents the baseline model, *κ* > 1 represents faster response, and *κ* < 1 represents slower response.

**Fig 12:**
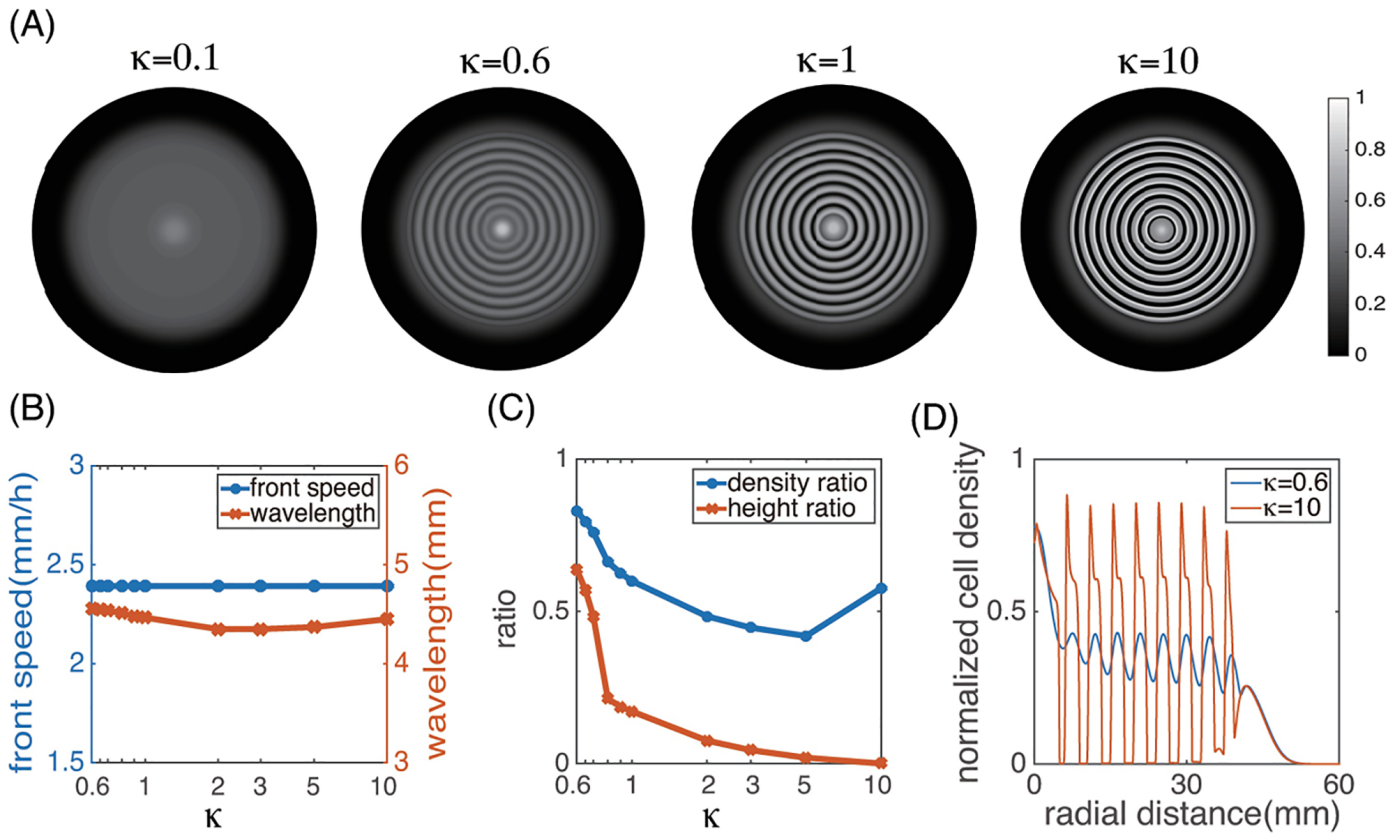
The cell density profile for different CheZ turnover rate *κ*. (A) Spatial density plots at *t* = 20h with *κ* = 0.1, 0.6, 1, 10, respectively. (B) Dependence of the front speed and wavelength on *κ*. (C) Dependence of the height ratio and density ratio on *κ*. (D) Plots of *ρ*(*ξ*, 20) for *κ* = 0.6 and 10. All other parameters are the same as in Fig 7.

Fig 12A plots the cell density profiles at *t* = 20h with *κ* = 0.1, 0.6, 1, 3 and 10. For *κ* = 0.1, CheZ concentration in cells does not change much over the whole computational time, and as a result cells remain highly mobile and form no stripes. As *κ* increases, the spatial stripes appear and become increasingly more prominent. Interestingly, both the colony front speed and the wavelength of the spatial pattern do not change much given different *κ* (Fig 12B). In contrast, the height ratio decreases to 0 as *κ* increases, due to the increase of the maximum cell density in each stripe and the decrease of the minimum cell density (Fig 12C); while the density ratio shows a biphasic dependence on *κ*: it first decreases significantly as the height ratio decreases and then increases as the height ratio becomes close to 0 and *κ* increases further. The rebound of the density ratio is primarily due to the widening of the high-density region in a stripe. Finally, we note that when *κ* is small, the peak in each stripe has a more or less symmetric shape (Fig 12D, blue); while as *κ* becomes large, the peak shows a higher density at locations with smaller radii (Fig 12D, red).

## Discussion

Synthetic biology has been used to design relatively simple systems to help understand how regularly-spaced structures form in nature. In [12], *E. coli* was engineered to couple chemotaxis and quorum sensing and these cells establish sequential stripe patterns when grown in semi-solid agar. In this paper, we developed multiscale models to help explain how these population patterns arise and predict their dependence on cell-level parameters.

We first developed a hybrid model that takes into account great details of intracellular signaling and movement of each individual cell. This model provides a method to connect cell-level dynamics and population-level behavior in a quantitative manner, but simulating it is very time-consuming as the cell number becomes large. To overcome this challenge, we mathematically derived a PDE model from our hybrid model. All the parameters of the PDE model can be calculated from measurable cell-level parameters used in the hybrid model. The PDE model matches the hybrid model quantitatively and is much more efficient in terms of computation. Our benchmark comparisons showed that the computation of the PDE model was over 100 times faster than that of the hybrid model. This justifies using the PDE model as a quantitative and predictive tool to explore the relation between population patterning and individual behavior.

Simulations of our models showed that the stripes arise sequentially due to suppression of CheZ in cells near the front of the expanding colony. At first, the self-secreted AHL reaches the threshold concentration for quorum sensing at these regions. This turns off the production of CheZ proteins in cells locally. The gradual drop of total CheZ inside these cells causes them to tumble excessively. As more and more cells move into these regions and get trapped, a high-density stripe develops. In the meantime, the colony grows and expands outward, and after some time, another high-density stripe establishes at a larger radius for the same reason. The self-trapping is due to the density-dependent suppression of motility, which has been studied before in [13, 25]. The model in [13] eliminates CheZ level by enslaving it to the AHL level, while the model in [25] directly links motility to the cell density, however both models are qualitative. The main contribution of our model is that it can not only reproduce the pattern, but also predict how the patterns varies when the individual cell signaling or movement changes. The spatial-temporal dynamics predicted by our simulations match experimental data *semi-quantitatively*.

We also made a number of predictions on the relation between the population patterns and cell level dynamics. Our simulations showed that the individual cell speed and the cell doubling time primarily affect the colony front speed and the wavelength of the stripe pattern (Fig 11). As the cell speed increases, the front speed and the pattern wavelength increases linearly. As the cell doubling time increases, the front speed decreases while the pattern wavelength increases. Moreover, the turnover rate of CheZ protein does not alter the colony front speed and pattern wavelength, but changes the spatial structure of each stripe characterized by the height ratio and density ratio (Fig 12). These predictions can be tested by further experiments.

Our PDE model gives a detailed characterization of the anisotropic movement of the whole cell population in response to AHL. Cells with different intracellular CheZ concentration *z* have different mobility coefficient, given by *D*(*z*) (Eq (18)). As a cell moves around, its internal state evolves with the extracellular environment. The change of *z* in each cell leads to the average mobility change of the whole population. We note that if *z* can be approximated by its steady state, which equals *Z*_*w*_ if *h* < *h*_0_ and 0 otherwise, then Eq (17) can be “formally” reduced to the anisotropic diffusion model used in [12]

$$\begin{array}{c} \partial_{t}\rho =\nabla_{x}\cdot (\nabla_{x}\left(\bar{D}\left(h\right)\rho \right))+rn\rho . \end{array}$$ (24)

where $\bar{D}$ is a step function of *h*. Specifically, we have *ρ*^*z*^(**x**, *t*, *z*) = *ρ*(**x**, *t*)*Q*(**x**, *t*, *z*) with

$$Q\left(x,t,z\right)=\{\begin{array}{cccc} & \delta (z-Z_{w}) & & h\left(x,t\right)<h_{0},\\ & \delta (z) & & h\left(x,t\right)\geq h_{0}. \end{array}$$

Integrating (17) with respect to *z*, one obtains Eq (24) with

$$\bar{D}\left(h\right)=\int D\left(z\right)Q\left(x,t,z\right)\;dz=\{\begin{array}{cccc} & D(Z_{w}) & & h<h_{0}\\ & D(0) & & h\geq h_{0}. \end{array}$$

However, during the stripe formation, CheZ turnover correlates with cell growth, which is much slower than single cell movement and intracellular signal adaptation. As a result, CheZ has a broad distribution among all cells and so it is far from its steady state (Fig 5). This suggest that it is important for models to take into account the internal state of cells individually rather than averaging it out.

We note that in this paper we used a multiscale modeling approach: start with a detailed, individual-based model for cell dynamics, then derive a PDE model and justify it using numerical simulations, and finally use the PDE model to make predictions on relations of phenomena at different scales. This multiscale approach allowed the macroscopic model to go beyond qualitative and can be used as a predictive tool. This type of multiscale modeling approach has also been used for classical bacterial chemotaxis [17, 21].

## Supporting information

S1 Text Parameter estimation for the turning rates. (PDF)

S2 Text Derivation of the PDE model. (PDF)

S3 Text Details of the numerical methods. (PDF)
