# Supplementary material for: The role of intracellular signaling in the stripe formation in engineered Escherichia coli populations
Source: PLoS Comput Biol. 2018 Jun 4;14(6):e1006178. doi: 10.1371/journal.pcbi.1006178 (PMC6002128; doi:10.1371/journal.pcbi.1006178)
Supplement: S1 Text — (PDF) [file pcbi.1006178.s001.pdf]

Supporting Information

Xiaoru Xue, Chuan Xue, Min Tang

S1: Parameter estimation for the turning rates

The transition rates λ_f and μ_f have been fitted in [1] using experimental data in Cluzel et al [2]. For the convenience of readers, we replotted the fitting graph in Figure S1. The dots are experimental data extracted from Fig. 2 in [2]. The lines are calculated using the formula (11) and (12).

Fig S1. The transition rates μ_f and λ_f. The red dots are experimental data extracted from Fig. 2 in [2]. The green lines are calculated using the formula.

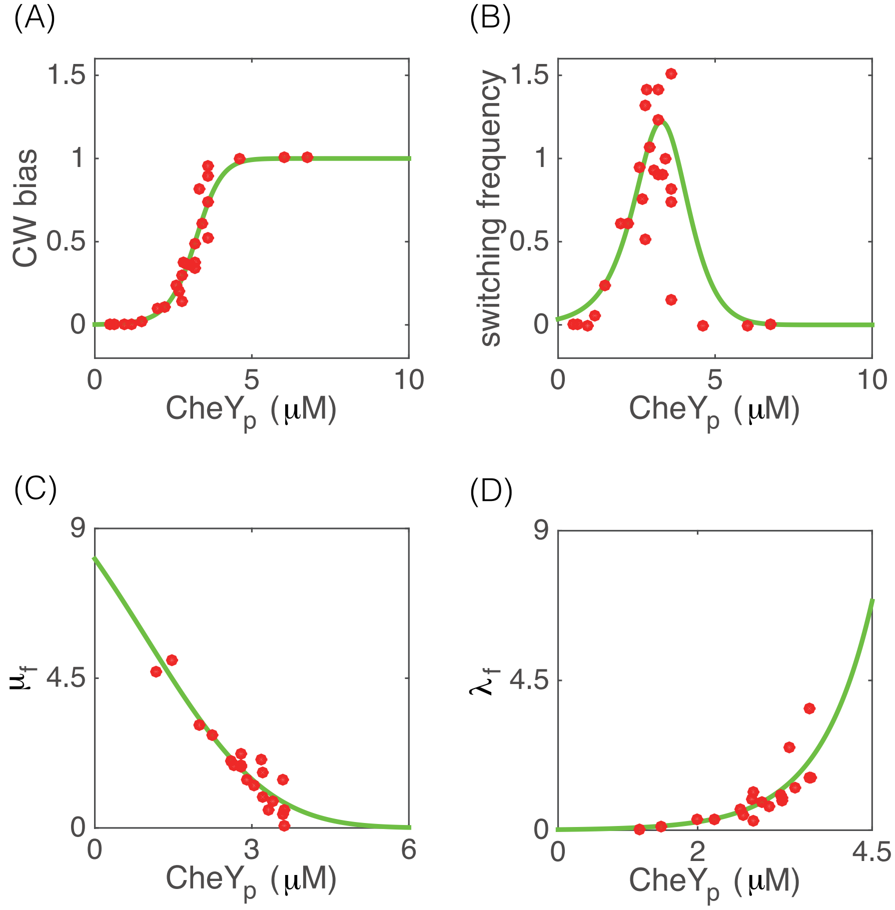

The turning rates λ and μ are determined by the voting process as described in the main text. In Table S1, we list the values of λ and μ under different choices of n_f, w and Z_w. The parameter values used in the numerical simulations are the same as e.g.4.

Table S1. Parameters for the voting process.

Param.	Description	e.g.1	e.g.2	e.g.3	e.g.4	e.g.5
n_f	total number of flagella in a cell	8	8	7	8	8
w	minimum number of CCW flagella required to propel the cell to run	7	6	6	6	6
$Z_w(\mu M)$	total CheZ protein in wild-type cells	1.3	1.25	1.25	1.23	1.2
$\lambda(s^{-1})$	transition rate from run to tumble	0.80	0.42	1.05	0.59	0.99
$\mu(s^{-1})$	transition rate from tumble to run	5.19	6.79	4.28	6.11	5.0
P_{run}	probability of running	0.87	0.94	0.80	0.91	0.84

References

1. Durney CH. A two-component model for bacterial chemotaxis. The Ohio State University; 2013.

2. Cluzel P, Surette M, Leibler S. An Ultrasensitive Bacterial Motor Revealed by Monitoring Signaling Proteins in Single Cells. Science. 2000;287:1652–1655.
