# Supplementary material for: The role of intracellular signaling in the stripe formation in engineered Escherichia coli populations
Source: PLoS Comput Biol. 2018 Jun 4;14(6):e1006178. doi: 10.1371/journal.pcbi.1006178 (PMC6002128; doi:10.1371/journal.pcbi.1006178)
Supplement: S2 Text — (PDF) [file pcbi.1006178.s002.pdf]

Supporting Information

Xiaoru Xue, Chuan Xue, Min Tang

S2: Derivation of the PDE model

In this appendix, we derive the continuous PDE model for the cell population dynamics, i.e., Eqns (17) and (18), from the cell-based component of the hybrid model, given by (15) and (16). We present the analysis in 1D in detail. The analysis in higher space dimensions is similar.

Eqns (17) and (18) in 1D can be rewritten as

$$\begin{aligned}
&\partial_t p^+ + s_0\partial_x p^+ + \partial_m(f(m,z)p^+) + \partial_z(g(z,h)p^+) = -\lambda(m,z)p^+ + \frac{\mu(m,z)}{2}p^0 + rnp^+,\\
&\partial_t p^- - s_0\partial_x p^- + \partial_m(f(m,z)p^-) + \partial_z(g(z,h)p^-) = -\lambda(m,z)p^- + \frac{\mu(m,z)}{2}p^0 + rnp^-,\\
&\partial_t p^0 + \partial_m(f(m,z)p^0) + \partial_z(g(z,h)p^0) = \lambda(m,z)(p^+ + p^-) - \mu(m,z)p^0 + rnp^0.
\end{aligned} \tag{S2.1}$$

Here $p^+(x,m,z,t)$, $p^-(x,m,z,t)$ and $p^0(x,m,z,t)$ are the density of cells that are moving to the right, to the left and stopping with internal state m and z at time t.

There are at least four intrinsic time scales enclosed in the above equations. The time scale for the stripe pattern formation (denoted by T). The time scale for the change of CheZ (denoted by T_1) is the same as the cell doubling time, which is approximately 30 minutes. The time scale for intracellular adaptation (denoted by T_2) is several seconds to a few minutes. The time scale for the run and tumble movement (denoted by T_3) is approximately $1s$.

We first nondimensionalize Eq (S2.1) by letting

$$t = T\tilde{t},\ x = L\tilde{x},\ s_0 = V_0\tilde{s}_0,\ z = Z\tilde{z},\ \mu = \frac{\tilde{\mu}}{T_3},\ \lambda = \frac{\tilde{\lambda}}{T_3},\ r = \frac{\tilde{r}}{T_1},$$

$$f(m,z) = \frac{\tilde{f}(m,z)}{T_2},\ g(z,h) = \frac{\tilde{g}(z,h)}{T_1},\ p^{\pm,0} = \tilde{p}^{\pm,0},$$

where T, L and V_0 are the characteristic temporal, spatial and velocity scales of the system respectively, and T_1, T_2 and T_3 are the characteristic doubling time, adaptation time and switching time respectively. After dropping the $\sim$, the nondimensionalized equations are follows:

$$\begin{aligned}
&\frac{1}{T}\partial_t p^+ + \frac{V_0}{L}s_0\partial_x p^+ + \frac{1}{T_2}\partial_m(f(m,z)p^+) + \frac{1}{T_1}\partial_z(g(z,h)p^+)\\
=&\frac{1}{T_3}\frac{\mu(m,z)}{2}p^0 - \frac{1}{T_3}\lambda(m,z)p^+ + \frac{1}{T_1}rnp^+,\\
&\frac{1}{T}\partial_t p^0 + \frac{1}{T_2}\partial_m(f(m,z)p^0) + \frac{1}{T_1}\partial_z(g(z,h)p^0)\\
=&\frac{1}{T_3}\lambda(m,z)(p^+ + p^-) - \frac{1}{T_3}\mu(m,z)p^0 + \frac{1}{T_1}rnp^0,\\
&\frac{1}{T}\partial_t p^- - \frac{V_0}{L}s_0\partial_x p^- + \frac{1}{T_2}\partial_m(f(m,z)p^-) + \frac{1}{T_1}\partial_z(g(z,h)p^-)\\
=&\frac{1}{T_3}\frac{\mu(m,z)}{2}p^0 - \frac{1}{T_3}\lambda(m,z)p^- + \frac{1}{T_1}rnp^-.
\end{aligned} \tag{S2.2}$$

All the quantities in Eq (S2.2) are nondimensionalized.

Moment closure in m:

Let

$$\rho^{\pm,0} = \int p^{\pm,0}\,\mathrm{d}m, \qquad q^{\pm,0} = \int m p^{\pm,0}\,\mathrm{d}m, \qquad M^{\pm,0} = q^{\pm,0}/\rho^{\pm,0}. \tag{S2.3}$$

Taking the integral of Eq (S2.2) with respect to m from 0 to m_{max}, one gets the equations for the zeroth-order moment:.

$$\begin{aligned}
&\frac{1}{T}\partial_t\rho^+ + \frac{V_0}{L}s_0\partial_x\rho^+ + \frac{1}{T_1}\partial_z(g(z,h)\rho^+)\\
=&\frac{1}{T_3}\int\frac{\mu(m,z)}{2}p^0\,\mathrm{d}m - \frac{1}{T_3}\int\lambda(m,z)p^+\,\mathrm{d}m + \frac{1}{T_1}rn\rho^+,\\
&\frac{1}{T}\partial_t\rho^0 + \frac{1}{T_1}\partial_z(g(z,h)\rho^0)\\
=&\frac{1}{T_3}\int\lambda(m,z)(p^+ + p^-)\,\mathrm{d}m - \frac{1}{T_3}\int\mu(m,z)p^0\,\mathrm{d}m + \frac{1}{T_1}rn\rho^0,\\
&\frac{1}{T}\partial_t\rho^- - \frac{V_0}{L}s_0\partial_x\rho^- + \frac{1}{T_1}\partial_z(g(z,h)\rho^-)\\
=&\frac{1}{T_3}\int\frac{\mu(m,z)}{2}p^0\,\mathrm{d}m - \frac{1}{T_3}\int\lambda(m,z)p^-\,\mathrm{d}m + \frac{1}{T_1}rn\rho^-.
\end{aligned} \tag{S2.4}$$

Then multiplying Eq (S2.2) by m and taking the integral with respect to m, one gets the equations for the first-order moment:

$$\begin{aligned}
&\frac{1}{T}\partial_t q^+ + \frac{V_0}{L}s_0\partial_x q^+ - \frac{1}{T_2}\int f(m,z)p^+\,\mathrm{d}m + \frac{1}{T_1}\partial_z(g(z,h)q^+)\\
=&\frac{1}{T_3}\int m\frac{\mu(m,z)}{2})p^0\,\mathrm{d}m - \frac{1}{T_3}\int m\lambda(m,z)p^+\,\mathrm{d}m + \frac{1}{T_1}rn\int mp^+\,\mathrm{d}m\\
&\frac{1}{T}\partial_t q^0 - \frac{1}{T_2}\int f(m,z)p^0\,\mathrm{d}m + \frac{1}{T_1}\partial_z(g(z,h)q^0)\\
=&\frac{1}{T_3}\int m\lambda(m,z)(p^+ + p^-)\,\mathrm{d}m - \frac{1}{T_3}\int m\mu(m,z)p^0\,\mathrm{d}m + \frac{1}{T_1}rn\int mp^0\,\mathrm{d}m,\\
&\frac{1}{T}\partial_t q^- - \frac{V_0}{L}s_0\partial_x q^- - \frac{1}{T_2}\int f(m,z)p^-\,\mathrm{d}m + \frac{1}{T_1}\partial_z(g(z,h)q^-)\\
=&\frac{1}{T_3}\int m\frac{\mu(m,z)}{2}p^0\,\mathrm{d}m - \frac{1}{T_3}\int m\lambda(m,z)p^-\,\mathrm{d}m + \frac{1}{T_1}rn\int mp^-\,\mathrm{d}m.
\end{aligned} \tag{S2.5}$$

Similar as in [25], we close the system (S2.4) and (S2.5) by making the assumption that m is locally concentrated. Denote

$$\lambda^\pm = \lambda(M^\pm, z), \qquad \mu^0 = \mu(M^0, z), \qquad F^{\pm,0} = f(M^{\pm,0}, z),$$

then from (S2.3), the terms in (S2.4) and (S2.5) can be approximated as follows:

$$\begin{aligned}
&\int\mu(m,z)p^0\,\mathrm{d}m \approx \int\Big(\mu(M^0,z) + \frac{\partial\mu}{\partial m}(M^0,z)\big(m - M^0\big)\Big)p^0\,\mathrm{d}m = \mu^0\rho^0,\\
&\int\lambda(m,z)p^\pm\,\mathrm{d}m \approx \int\Big(\lambda(M^\pm,z) + \frac{\partial\lambda}{\partial m}(M^\pm,z)\big(m - M^\pm\big)\Big)p^\pm\,\mathrm{d}m = \lambda^\pm\rho^\pm,\\
&\int f(m,z)p^{\pm,0}\,\mathrm{d}m \approx \int\Big(f(M^{\pm,0},z) + \frac{\partial f}{\partial m}(M^{\pm,0},z)\big(m - M^{\pm,0}\big)\Big)p^\pm\,\mathrm{d}m = F^{\pm,0}\rho^{\pm,0},\\
&\int m\lambda(m,z)p^\pm\,\mathrm{d}m \approx \int\Big(M^\pm\lambda(M^\pm,z) + \frac{\partial(m\lambda)}{\partial m}(M^\pm,z)\big(m - M^\pm\big)\Big)p^\pm\,\mathrm{d}m\\
&\qquad\qquad = M^\pm\lambda^\pm\rho^\pm = \lambda^\pm q^\pm.
\end{aligned}$$

Therefore, the closed moment system becomes:

$$\begin{aligned}
&\frac{1}{T}\partial_t\rho^+ + \frac{V_0}{L}s_0\partial_x\rho^+ + \frac{1}{T_1}\partial_z(g(z,h)\rho^+) = \frac{1}{T_3}\frac{\mu^0}{2}\rho^0 - \frac{1}{T_3}\lambda^+\rho^+ + \frac{1}{T_1}rn\rho^+,\\
&\frac{1}{T}\partial_t\rho^0 + \frac{1}{T_1}\partial_z(g(z,h)\rho^0) = \frac{1}{T_3}\lambda^+\rho^+ + \frac{1}{T_3}\lambda^-\rho^- - \frac{1}{T_3}\mu^0\rho^0 + \frac{1}{T_1}rn\rho^0,\\
&\frac{1}{T}\partial_t\rho^- - \frac{V_0}{L}s_0\partial_x\rho^- + \frac{1}{T_1}\partial_z(g(z,h)\rho^-) = \frac{1}{T_3}\frac{\mu^0}{2}\rho^0 - \frac{1}{T_3}\lambda^-\rho^- + \frac{1}{T_1}rn\rho^-.
\end{aligned} \tag{S2.6}$$

$$\begin{aligned}
&\frac{1}{T}\partial_t q^+ + \frac{V_0}{L}s_0\partial_x q^+ - \frac{1}{T_2}F^+\rho^+ + \frac{1}{T_1}\partial_z(g(z,h)q^+) = \frac{1}{T_3}\frac{\mu^0}{2}q^0 - \frac{1}{T_3}\lambda^+ q^+ + \frac{1}{T_1}rnq^+,\\
&\frac{1}{T}\partial_t q^0 - \frac{1}{T_2}F^0\rho^0 + \frac{1}{T_1}\partial_z(g(z,h)q^0) = \frac{1}{T_3}\lambda^+ q^+ + \frac{1}{T_3}\lambda^- q^- - \frac{1}{T_3}\mu^0 q^0 + \frac{1}{T_1}rnq^0,\\
&\frac{1}{T}\partial_t q^- - \frac{V_0}{L}s_0\partial_x q^- - \frac{1}{T_2}F^-\rho^- + \frac{1}{T_1}\partial_z(g(z,h)q^-) = \frac{1}{T_3}\frac{\mu^0}{2}q^0 - \frac{1}{T_3}\lambda^- q^- + \frac{1}{T_1}rnq^-.
\end{aligned} \tag{S2.7}$$

Asymptotic analysis:

We take the parabolic scaling such that:

$$\frac{T}{L/V_0} = \frac{1}{\epsilon}, \quad \frac{T_1}{L/V_0} = \frac{1}{\epsilon}, \quad \frac{T_2}{L/V_0} = 1, \quad \frac{T_3}{L/V_0} = \epsilon \tag{S2.8}$$

Here ϵ is very small. In the experiment, this scaling is valid. First of all, we choose the characteristic time of the system the same as the cell doubling time T_1 which is $20 \sim 40$ minutes, while the adaptation time T_2 is around $10 \sim 100$ seconds and the switching time between the running and tumbling states T_3 is around $1s$. The value of ϵ can be determined by the second and fourth equations in (S2.8) such that $\epsilon^2 = T_3/T_1$. We take $T_3 = 1s$ and let $\epsilon = 0.025$, then $T_1 = 26.67mins$ which belongs to the interval of $20 \sim 40$ minutes. The typical space scale is $L = 1mm$ and according to the values of T_3 and ϵ, we take the characteristic velocity V_0 to be $0.025mm/s$. Similarly, we can determine $T = 26.67mins$ and the characteristic adaptation time $T_2 = 40s$.

Plugging (S2.8) into (S2.6) and (S2.7) yields:

$$\begin{aligned}
&\epsilon\partial_t\rho^+ + s_0\partial_x\rho^+ + \epsilon\partial_z(g(z,h)\rho^+) = \frac{1}{\epsilon}\frac{\mu^0}{2}\rho^0 - \frac{1}{\epsilon}\lambda^+\rho^+ + \epsilon rn\rho^+,\\
&\epsilon\partial_t\rho^0 + \epsilon\partial_z(g(z,h)\rho^0) = \frac{1}{\epsilon}\lambda^+\rho^+ + \frac{1}{\epsilon}\lambda^-\rho^- - \frac{1}{\epsilon}\mu^0\rho^0 + \epsilon rn\rho^0,\\
&\epsilon\partial_t\rho^- - s_0\partial_x\rho^- + \epsilon\partial_z(g(z,h)\rho^-) = \frac{1}{\epsilon}\frac{\mu^0}{2}\rho^0 - \frac{1}{\epsilon}\lambda^-\rho^- + \epsilon rn\rho^-.
\end{aligned} \tag{S2.9}$$

$$\begin{aligned}
&\epsilon\partial_t q^+ + s_0\partial_x q^+ - F^+\rho^+ + \epsilon\partial_z(g(z,h)q^+) = \frac{1}{\epsilon}\frac{\mu^0}{2}q^0 - \frac{1}{\epsilon}\lambda^+ q^+ + \epsilon rnq^+,\\
&\epsilon\partial_t q^0 - F^0\rho^0 + \epsilon\partial_z(g(z,h)q^0) = \frac{1}{\epsilon}\lambda^+ q^+ + \frac{1}{\epsilon}\lambda^- q^- - \frac{1}{\epsilon}\mu^0 q^0 + \epsilon rnq^0,\\
&\epsilon\partial_t q^- - s_0\partial_x q^- - F^-\rho^- + \epsilon\partial_z(g(z,h)q^-) = \frac{1}{\epsilon}\frac{\mu^0}{2}q^0 - \frac{1}{\epsilon}\lambda^- q^- + \epsilon rnq^-.
\end{aligned} \tag{S2.10}$$

Assume that the solution to the above system has the following asymptotic expansion

$$\rho^{\pm,0} = \rho_0^{\pm,0} + \epsilon\rho_1^{\pm,0} + \epsilon^2\rho_2^{\pm,0} + \cdots, \qquad q^{\pm,0} = q_0^{\pm,0} + \epsilon q_1^{\pm,0} + \epsilon^2 q_2^{\pm,0} + \cdots,$$

By matching different orders, one gets, at $O(1/\epsilon)$

$$\frac{\mu_0^0}{2}\rho_0^0 = \lambda_0^+\rho_0^+, \quad \lambda_0^+\rho_0^- + \lambda_0^+\rho_0^- = \mu_0^0\rho_0^0, \quad \frac{\mu_0^0}{2}\rho_0^0 = \lambda_0^-\rho_0^-,$$
$$\frac{\mu_0^0}{2}q_0^0 = \lambda_0^+q_0^+, \quad \lambda_0^+q_0^- + \lambda_0^+q_0^- = \mu_0^0q_0^0, \quad \frac{\mu_0^0}{2}q_0^0 = \lambda_0^-q_0^-.$$

Thus

$$\lambda_0^+\rho_0^+ = \lambda_0^-\rho_0^- = \frac{\mu_0^0}{2}\rho_0^0, \qquad M_0^+ = M_0^- = M_0^0.$$

We then use the following notations:

$$M_0^+ = M_0^- = M_0^0 = M_0, \quad \lambda_0^+ = \lambda_0^- = \lambda_0, \quad \rho_0^+ = \rho_0^- = \frac{\mu_0^0}{2\lambda_0}\rho_0^0 = \rho_0. \tag{S2.11}$$

At $O(1)$,

$$\begin{aligned}
s_0\partial_x\rho_0 &= \frac{\mu_0^0}{2}\rho_1^0 + \frac{\mu_1^0}{2}\rho_0^0 - \lambda_1^+\rho_0 - \lambda_0\rho_1^+,\\
-s_0\partial_x\rho_0 &= \frac{\mu_0^0}{2}\rho_1^0 + \frac{\mu_1^0}{2}\rho_0^0 - \lambda_1^-\rho_0 - \lambda_0\rho_1^-,\\
\lambda_0\rho_1^+ + \lambda_1^+\rho_0 + \lambda_0\rho_1^- + \lambda_1^-\rho_0 &= \mu_0^0\rho_1^0 + \mu_1^0\rho_0^0,\\
s_0\partial_x q_0 - F_0\rho_0 &= \frac{\mu_0^0}{2}q_1^0 + \frac{\mu_1^0}{2}q_0^0 - \lambda_1^+q_0 - \lambda_0q_1^+,\\
-s_0\partial_x q_0 - F_0\rho_0 &= \frac{\mu_0^0}{2}q_1^0 + \frac{\mu_1^0}{2}q_0^0 - \lambda_1^-q_0 - \lambda_0q_1^-,\\
-F_0\rho_0^0 &= \lambda_0q_1^+ + \lambda_1^+q_0 + \lambda_0q_1^- + \lambda_1^-q_0 - \mu_0^0q_1^0 - \mu_1^0q_0^0.
\end{aligned} \tag{S2.12}$$

Let $\bar\rho = \rho^+ + \rho^- + \rho^0$, the summation of the last three equations in Eq (S2.12) yields $F_0\bar\rho_0 = 0$, which indicates that

$$\bar\rho_0(x,t,m,z) = \big(\int \bar\rho_0(x,t,m,z)\,\mathrm{d}m\big)\delta\big(m - M_0(m_0,z)\big) = \rho^z(x,t,z)\delta\big(m - M_0(m_0,z)\big),$$

with $\rho^z \equiv \int \bar\rho_0(x,t,m,z)\,\mathrm{d}m$ and

$$k_R R\Big(1 - A(M_0(m_0,z))\Big) - k_{Bp}B_pA(M_0(m_0,z)) = 0 \tag{S2.13}$$

from Eq (2). Here M_0 is a function of m_0 and z determined implicitly by the system (5) together with Eq (S2.13). Then from $\lambda^{\pm,0} = \lambda(M^{\pm,0},z)$, $\lambda_0^{\pm,0} = \lambda(M_0^{\pm,0},z) = \lambda(M_0,z)$ is a function of m_0 and z as well. Note that $\bar\rho_0$ is different from ρ_0, while from (S2.11), they are connected by

$$\bar\rho_0 = 2\rho_0 + \frac{2\lambda_0}{\mu_0^0}\rho_0 = \big(2 + \frac{2\lambda_0}{\mu_0^0}\big)\rho_0,$$

thus we have

$$\rho_0(x,t,m,z) = \big(\int \rho_0(x,t,m,z)\,\mathrm{d}m\big)\delta(m - M_0(m_0,z)) = \tilde\rho_0(x,t,z)\delta\big(m - M_0(m_0,z)\big). \tag{S2.14}$$

The subtraction of the first two equations and the subtraction of the forth and fifth equations in Eq (S2.12) give

$$\begin{aligned}
-2s_0\partial_x\rho_0 &= \rho_0(\lambda_1^+ - \lambda_1^-) + \lambda_0(\rho_1^+ - \rho_1^-),\\
-2s_0\partial_x q_0 &= q_0(\lambda_1^+ - \lambda_1^-) + \lambda_0M_0(\rho_1^+ - \rho_1^-) + \lambda_0\rho_0(M_1^+ - M_1^-).
\end{aligned}$$

Thanks to Eq (S2.14), taking the integral with respect to m of the above two equations yields

$$-2s_0\partial_x\tilde{\rho}_0 = \tilde{\rho}_0\big(\lambda_1^+ - \lambda_1^-\big) + \lambda_0\int\big(\rho_1^+ - \rho_1^-\big)\,\mathrm{d}m,$$
$$-2s_0\partial_x\big(M_0\tilde{\rho}_0\big) = M_0\tilde{\rho}_0\big(\lambda_1^+ - \lambda_1^-\big) + M_0\lambda_0\int\big(\rho_1^+ - \rho_1^-\big)\,\mathrm{d}m + \lambda_0\tilde{\rho}_0\big(M_1^+ - M_1^-\big).$$

Since M_0 is determined by (S2.13) together with Eq (5), which is independent of x,

$$\lambda_0\tilde{\rho}_0(M_1^+ - M_1^-) = -2s_0\tilde{\rho}_0\partial_x M_0 = 0.$$

Besides from

$$\lambda_1^+ - \lambda_1^- = \frac{1}{\epsilon}\big(\lambda(M_0 + \epsilon M_1^+) - \lambda(M_0 + \epsilon M_1^-) + O(\epsilon^2)\big) = O(\epsilon)$$

we have

$$\int\big(\rho_1^+ - \rho_1^-\big)\,\mathrm{d}m = -2s_0\lambda_0^{-1}\partial_x\tilde{\rho}_0,$$

The limiting macroscopic equation can be obtained by considering the $O(\epsilon)$ terms in the summation of the three equations in Eq (S2.9):

$$\partial_t\bar{\rho}_0 + s_0\partial_x(\rho_1^+ - \rho_1^-) + \partial_z(g(z,h)\bar{\rho}_0) = rn\bar{\rho}_0.$$

Then from $\bar{\rho} = \rho^+ + \rho^- + \rho^0$ and Eq (S2.11), the macroscopic total density $\rho^z = \big(2 + 2\frac{\lambda_0}{\mu_0}\big)\tilde{\rho}_0$ satisfies

$$\partial_t\rho^z = \partial_x\big(D(z)\partial_x\rho^z\big) - \partial_z(g(z,h)\rho^z) + rn\rho^z, \tag{S2.15}$$

with z-dependent diffusion coefficients $D(z) = \frac{s_0^2}{1+\lambda_0/\mu_0}\lambda_0^{-1}$.

Extension to higher dimensional case is straightforward and the diffusion coefficient $D(z)$ becomes

$$D(z) = \frac{s_0^2\mu_0(z)}{d\lambda_0(z)\Big[\mu_0(z) + \lambda_0(z)\Big]}.$$
