# Supplementary material for: The role of intracellular signaling in the stripe formation in engineered Escherichia coli populations
Source: PLoS Comput Biol. 2018 Jun 4;14(6):e1006178. doi: 10.1371/journal.pcbi.1006178 (PMC6002128; doi:10.1371/journal.pcbi.1006178)
Supplement: S3 Text — (PDF) [file pcbi.1006178.s003.pdf]

Supporting Information

Xiaoru Xue, Chuan Xue, Min Tang

S3: Details of the numerical methods

Numerical method for the hybrid model

The numerical method for the hybrid model is similar to the one used in [22]. In the following, we give the details of the method in 1D.

We took the computational domain to be $[-40mm, 40mm]$. We represented each cell by its position x^i, internal methylation level m^i, internal CheZ concentration z^i and velocity s^i, where the superscript i is the index of the cell. We solved Eqns (14) for the nutrient and AHL using finite difference method with space step $\Delta x = 0.1$mm and time step $\Delta t = 0.1$s. Here we denote the numerical approximations of $n(j\Delta x, k\Delta t)$ and $h(j\Delta x, k\Delta t)$ by n_j^k and h_j^k.

Initialization We randomly put 500 cells in the domain according to the distribution

$$P(x) = \frac{1}{\sigma\sqrt{2\pi}} \exp\Big(-\frac{x^2}{2\sigma^2}\Big)$$

with $\sigma = 2$mm. We assume that initially the internal state of these cells is at equilibrium, with $z = 1.23\mu$M and $m = 0.7071$. The initial velocity of a cell is randomly selected from 0 (tumbling), s_0 (right-moving) and $-s_0$ (left-moving) with equal probability. For the nutrient and AHL, we took $n(x, 0) = 1$, $h(x, 0) = 0$.

Time evolution For each time step k (= 1 initially), we perform the following calculations:

a) Update cell data.

- Update internal states. Update z^i and m using (1) and (2). The AHL concentration h along the cell trajectory is linearly interpolated from the values at the grid points. R, B_p are first solved from the algebraic system (5), using the built-in MATLAB function "fsolve" with error tolerances 10^{-10}. The ODEs are solved using the built-in MATLAB solver "ode113" with error tolerances 10^{-10}. The obtained $\mathrm{CheY_p}$ level in the second step is used to update λ^i and μ^i.
- Update cell velocity. For each tumbling cell, generate two random numbers r_1 and r_2 uniformly distributed in $[0, 1]$. If $r_1 \leq \mu^i\Delta t$, then update cell velocity using the following method: if $r_2 \leq 0.5$ then set the cell velocity to s_0, otherwise $-s_0$. For each moving cell, if $r_1 \leq \lambda^i\Delta t$, then set the cell speed to 0.
- Update cell position. Resets cell position to $x^i + v^i\Delta t$ where v^i is the current velocity of the cell.
- Update cell population due to cell division. For each cell, generate a random number r_3 uniformly distributed in $[0, 1]$. If $r_3 \leq rNdt$, then replace the cell by two exact copies of itself. Here N is the nutrient concentration at the nearest neighbor grid point.

b) Update the AHL and nutrient concentrations. First estimate the cell density ρ_j in the interval $[(j-1/2)\Delta x, (j+1/2)\Delta x]$ in terms of number of cells per unit length in the interval. Then use the following finite difference method:

$$\frac{h_i^{k+1}-h_i^k}{\Delta t} = D_h \frac{h_{i+1}^{k+1}-2h_i^{k+1}+h_{i-1}^{k+1}}{\Delta x^2} + \alpha_d \rho_j^k - \beta h_i^{k+1},$$
$$\frac{n_i^{k+1}-n_i^k}{\Delta t} = D_n \frac{n_{i+1}^{k+1}-2n_i^{k+1}+\rho_{i-1}^{k+1}}{\Delta x^2} - \gamma_d \rho_j^k n_i^{k+1}.$$

b) $k \leftarrow k+1$. If $k \times \Delta > T_0$ stop.

Numerical algorithm for the PDE model

For spatial discretization, we used central difference for the diffusion operator and upwind scheme for the advection term in z. We defined ghost points to treat the Neumann boundary conditions in x for ρ, h and n. For temporal discretization, we used semi-implicit scheme.

Consider the computational domain $(x,z) \in [a,b] \times [0, Z_w]$. Denote the uniform mesh size for x, z and t by Δx, Δz and Δt. Define $x_i = a + i\Delta x$, $z_j = j\Delta z$ and $t^k = k\Delta t$. Denote the numerical solution by $\rho_{i,j}^k \approx \rho^z(x_i, z_j, t^k)$, $n_i^k \approx n(x_i, t^k)$ and $h_i^k \approx h(x_i, t^k)$.

From the definition of $g(z,h)$ in (1), the sign of $g(z,h)$ only depends on h. Thus we used the following discretization for Eqn (17)

$$\begin{aligned}
\frac{\rho_{i,j}^{k+1}-\rho_{i,j}^k}{\Delta t} =& D(z_j)\frac{\rho_{i+1,j}^{k+1}-2\rho_{i,j}^{k+1}+\rho_{i-1,j}^{k+1}}{\Delta x^2} + rn_i^k \rho_{i,j}^{k+1} \\
&- \frac{g(z_j,h_i^k)\rho_{i,j}^k - g(z_{j-1},h_i^k)\rho_{i,j-1}^k}{\Delta z}, \qquad \text{for } g(z_j,h_i^k) > 0, \\
\frac{\rho_{i,j}^{k+1}-\rho_{i,j}^k}{\Delta t} =& D(z_j)\frac{\rho_{i+1,j}^{k+1}-2\rho_{i,j}^{k+1}+\rho_{i-1,j}^{k+1}}{\Delta x^2} + rn_i^k \rho_{i,j}^{k+1} \\
&- \frac{g(z_{j+1},h_i^k)\rho_{i,j+1}^k - g(z_j,h_i^k)\rho_{i,j}^k}{\Delta z}, \qquad \text{for } g(z_j,h_i^k) < 0.
\end{aligned}$$

Neumann boundary conditions are used for space and CheZ, their discretizations write

$$\begin{aligned}
&\rho_{0,j}^{k+1} = \rho_{1,j}^{k+1}, \qquad \rho_{N_x-1,j}^{k+1} = \rho_{N_x,j}^{k+1}, \qquad \forall j; \\
&\rho_{i,0}^{k+1} = \rho_{i,1}^{k+1}, \qquad \text{for } g(z_j,h_i^k) > 0, \qquad \rho_{i,N_z-1}^{k+1} = \rho_{i,N_z}^{k+1}, \qquad \text{for } g(z_j,h_i^k) < 0.
\end{aligned}$$

The discretization for the system (19) is given by

$$\frac{h_i^{k+1}-h_i^k}{\Delta t} = D_h \frac{h_{i+1}^{k+1}-2h_i^{k+1}+h_{i-1}^{k+1}}{\Delta x^2} + \alpha \sum_{j=1}^{N_z} \frac{\Delta z}{2}(\rho_{i,j}^k + \rho_{i,j+1}^k) - \beta h_i^{k+1},$$

$$\frac{n_i^{k+1}-n_i^k}{\Delta t} = D_n \frac{n_{i+1}^{k+1}-2n_i^{k+1}+\rho_{i-1}^{k+1}}{\Delta x^2} - \gamma n_i^{k+1} \sum_{j=1}^{N_z} \frac{\Delta z}{2}(\rho_{i,j}^k + \rho_{i,j+1}^k).$$

Neumann boundary conditions are used for h, n and their discretizations write

$$h_0^{k+1} = h_1^{k+1}, \qquad h_{N_x-1}^{k+1} = h_{N_x}^{k+1}, \qquad n_0^{k+1} = n_1^{k+1}, \qquad n_{N_x-1}^{k+1} = n_{N_x}^{k+1}.$$
